# *D2HGDH* Deficiency Regulates Seizures through GSH/Prdx6/ROS‐Mediated Excitatory Synaptic Activity

**DOI:** 10.1002/advs.202404488

**Published:** 2024-12-30

**Authors:** Zhijuan Zhang, Hui Zhang, Peng Zhang, Rong Li, Jinyu Zhou, Jiyuan Li, Danmei Hu, Rui Huang, Fenglin Tang, Jie Liu, Demei Xu, Chenlu Zhang, Xin Tian, Yuanlin Ma, Patrick Kwan

**Affiliations:** ^1^ Department of Neurology The First Affiliated Hospital of Chongqing Medical University Chongqing Key Laboratory of Neurology Chongqing 400016 China; ^2^ Department of Neurology The Second Affiliated Hospital of Chongqing Medical University Chongqing Key Laboratory of Neurology Chongqing 400010 China; ^3^ Department of Neurology The First People's Hospital of Yunnan Province The Affiliated Hospital of Kunming University of Science and Technology Kunming Yunnan 650032 China; ^4^ Yunnan Provincial Key Laboratory for Birth Defects and Genetic Diseases First People's Hospital of Yunnan Province Kunming 650051 China; ^5^ Department of Neurology The First Hospital of Shanxi Medical University Shanxi 030012 China; ^6^ Department of Neurology Shanxi Bethune Hospital Shanxi 030032 China; ^7^ Key Laboratory of Major Brain Disease and Aging Research (Ministry of Education) Chongqing Medical University Chongqing 400016 China; ^8^ Department of Neuroscience Central Clinical School Monash University Melbourne 3004 Australia

**Keywords:** D‐2‐hydroxyglutarate dehydrogenase, epilepsy, excitatory synapses, reactive oxygen species

## Abstract

Current antiepileptic drugs are ineffective in one‐third of patients with epilepsy; however, identification of genes involved in epilepsy can enable a precision medicine approach. Here, it is demonstrated that downregulating D‐2‐hydroxyglutarate dehydrogenase (*D2HGDH*) enhances susceptibility to epilepsy. Furthermore, its potential involvement in the seizure network through synaptic function modulation is investigated. *D2HGDH* knockdown reduces the glutathione reduced (GSH)/glutathione oxidized (GSSG) ratio and elevates reactive oxygen species (ROS) levels within neurons. Oxidative stress may play a crucial role in the pathogenesis of epilepsy. The specific contribution of each pathway varies among patients, highlighting the complexity of this disease. In this study, downregulation of *D2HGDH* affects modulation of ROS levels, synaptic transmission, and seizure susceptibility. Furthermore, the acid calcium‐independent phospholipase A2 (aiPLA2) inhibitor, MJ33, restores the GSH/GSSG balance and reverses the increase in ROS levels caused by *D2HGDH* knockdown, resulting in remission of epilepsy‐related behaviors. The results demonstrate that downregulation of *D2HGDH* affects synaptic function by regulating ROS production. These findings support the use of targeted gene therapy as a potential alternative to antioxidant‐based treatments for refractory epilepsy.

## Graphical abstract

Downregulating D‐2‐hydroxyglutarate dehydrogenase (*D2HGDH*) increases epilepsy susceptibility by altering reactive oxygen species (ROS) levels and synaptic function, contributing to oxidative stress in neurons. The acid calcium‐independent phospholipase A2 (aiPLA2) inhibitor MJ33 restores glutathione reduced/glutathione oxidized balance, reducing ROS and improving epilepsy‐related behaviors. These findings highlight *D2HGDH* as a potential target for gene therapy, offering a precision medicine approach for refractory epilepsy.

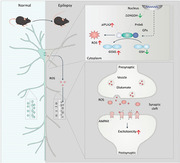

## 1 Introduction

Epilepsy, characterized by recurrent, unprovoked seizures, affects ≈70 million people worldwide.^[^ ^1^ ^]^ Epilepsy has been linked to hundreds of genes with varied biological functions, including those encoding ion channel subunits, transcription factors, and metabolic enzymes.^[^ ^2^ ^]^ Anti‐seizure medications (ASMs), which reduce neuronal excitation or enhance neuronal inhibition, are the primary treatments for epilepsy. However, current anti‐seizure medications are ineffective in one‐ third of patients^[^ ^3^ ^]^ and act indiscriminately throughout the nervous system, leading to a wide range of adverse effects.^[^ ^4^ ^]^ However, the mechanisms underlying pharmaco‐resistance in epilepsy remain unclear.^[^ ^5^ ^]^ One study found that serum antioxidant status was lower in patients with epilepsy than in healthy controls;^[^ ^6^ ^]^ Interestingly, antioxidant compounds may demonstrate beneficial effects when used in conjunction with ASMs, suggesting an association between the epileptic phenotype and oxidative stress content.^[^ ^7^ ^]^

d‐2‐hydroxyglutarate dehydrogenase (*D2HGDH*) is the primary enzyme responsible for the catabolism of D‐2‐hydroxyglutarate (D‐2‐HG).^[^ ^8^ ^]^ Numerous clinical reports have shown that downregulation of *D2HGDH* causes elevated D‐2‐HG levels,^[^ ^9^ ^]^ and *D2HGDH* mutations are associated with neonatal epileptic encephalopathy, hypotension, delayed visual brain development, cardiomyopathy, and facial dysmorphia.^[^ ^10^ ^]^ Reduction in *D2HGDH* protein expression by ≈50% can lead to metabolic and cellular consequences that support a haploinsufficiency model for heterozygous inactivating mutations in this gene.^[^ ^11^ ^]^ Detailed enzymatic and cellular analyses have characterized these mutations as loss‐of‐function mutations,^[^ ^12^ ^]^ suggesting that accumulation of ROS may contribute to the pathogenesis of disorders associated with *D2HGDH* loss.^[^ ^10b^ ^]^ L2HG offers a novel mechanism for eliminating ROS from myocardial tissues, mitigating redox stress, reducing myocardial infarct size, and preserving high‐energy phosphate levels and cardiac function.^[^ ^13^ ^]^ Furthermore, seizures have been described in 20%–35% of patients with deletions of or within the 2q37 locus, a frequently deleted subtelomeric region containing 197 genes.^[^ ^14^ ^]^ A recent genotype–phenotype correlation analysis of 2q37 deletions identified *HDAC4* and *D2HGDH* as candidate genes for the treatment of seizures,^[^ ^15^ ^]^ and patients with *D2HGDH* deletions have been reported to have seizures and neurodevelopmental abnormalities.^[^ ^9^ ^]^ However, the molecular mechanisms underlying this association are poorly understood.

To elucidate the potential role and mechanism of *D2HGDH* in epilepsy, this study 1) determined the localization of *D2HGDH* in human and C57BL/6J brain tissues; 2) investigated the potential involvement of *D2HGDH* in epilepsy models, including kainic acid (KA) and pentylenetetrazol (PTZ) models; 3) assessed the functional effects of *D2HGDH* on hippocampal neurons in C57BL/6J mice; and 4) used metabolomics, proteomics, and gene set enrichment analysis (GSEA) to explore potential downstream pathways following downregulation of *D2HGDH*.

Behavioral observations, western blotting, immunofluorescence staining, and electrophysiological methods were used to investigate the influence of *D2HGDH* on epilepsy and its underlying mechanisms. Overall, this study aimed to investigate how *D2HDGH* contributes to neuronal functional metabolism and regulates synaptic activity through ROS production, thereby shedding light on the pathogenesis underlying its effects on epilepsy.

## 2 Results

### 2.1 Expression of *D2HGDH* in Epileptic Patients’ Brains and Localization in C57BL/6J Mice

**Figure 1:**
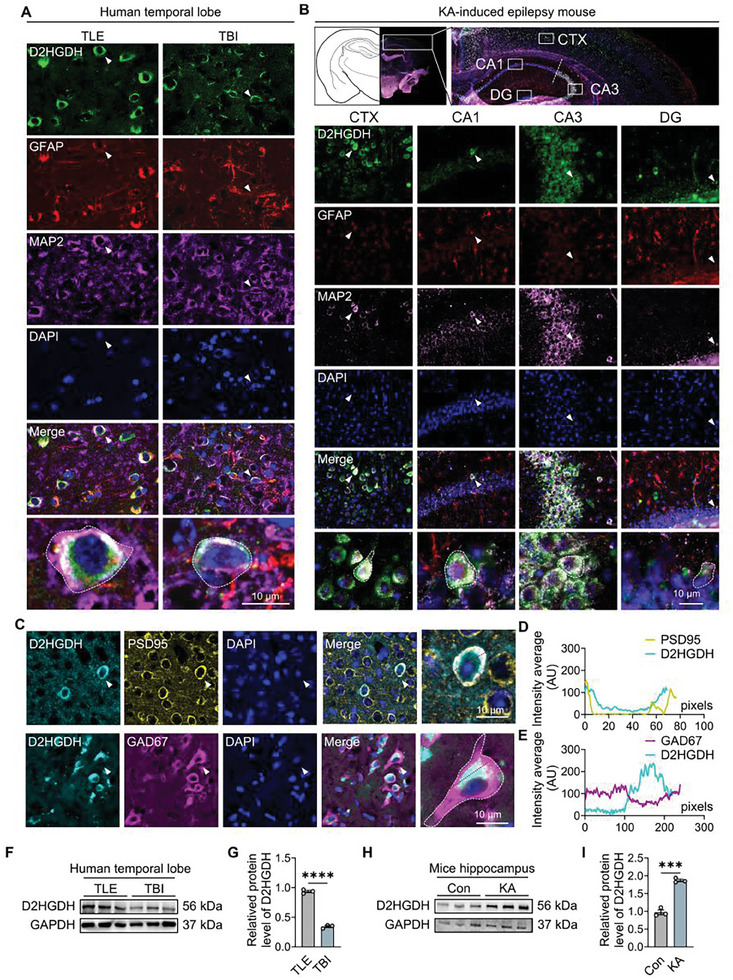
Expression and localization of *D2HGDH* in epileptic brain tissues. A) Co‐staining of *D2HGDH*, GFAP, and MAP2 in human temporal lobe tissues, comparing TLE and TBI; scale bar as indicated. B) Co‐staining of *D2HGDH*, GFAP, and MAP2 in a mouse model of KA‐induced epilepsy; the hippocampus, including CA1, CA3, DG, and cortex (CTX); scale bar as indicated. C) Co‐staining of *D2HGDH*, PSD95, and GAD67 in human temporal lobe tissues; scale bar as indicated. D,E) Fluorescence co‐localization intensity curve of (C). F) Representative images of human temporal lobe proteins in the TLE group (*n* = 3) versus the TBI group (*n* = 3). G) Quantized data for protein levels of *D2HGDH* in the TLE group (*n* = 3) versus the TBI group (*n* = 3), analyzed by Student's *t*‐test. H) Representative images of mice hippocampal proteins in the control group (*n* = 3) versus the KA group (*n* = 3). I) Quantized data for mice hippocampal protein levels of *D2HGDH* in the control group (*n* = 3) versus the KA group (*n* = 3), analyzed using Student's *t*‐test.

Because *D2HGDH* is strongly linked to epilepsy,^[^ ^16^ ^]^ we used human brain tissue from patients with temporal lobe epilepsy (TLE) (see Table S1, Supporting Information, for details) and tissue from C57BL/6J mice to investigate the precise localization and expression patterns of this gene (**Figure** **1**A,B). Although *D2HGDH* is abundantly expressed in the brain tissue, its distribution in the hippocampus remains unclear. Immunofluorescence staining of slices from C57BL/6J mouse brains demonstrated a widespread distribution of *D2HGDH* within the hippocampus, including the CA1, CA3, and dentate gyrus (DG) areas, and the cortex (Figure S1, Supporting Information; Figure 1B).

First, analysis of epileptic brain tissue revealed that *D2HGDH* was predominantly localized within neurons (Figure 1A), with elevated expression observed specifically in patients with TLE compared with that in patients with traumatic brain injury (TBI) (Figure 1A,F,G). Second, the same results were obtained in the KA‐induced epilepsy model (Figure 1B,H,I).

Notably, abundant co‐localization was observed primarily with MAP2 rather than GFAP (Figure 1A,B; Figure S1, Supporting Information) and primarily with PSD95 rather than GAD67 (Figure 1C–E); this has not been previously investigated. This pattern indicates a potentially crucial role of *D2HGDH* in epileptic disorders.

### 2.2 *D2HGDH* Modulates Seizure Susceptibility and Severity

**Figure 2:**
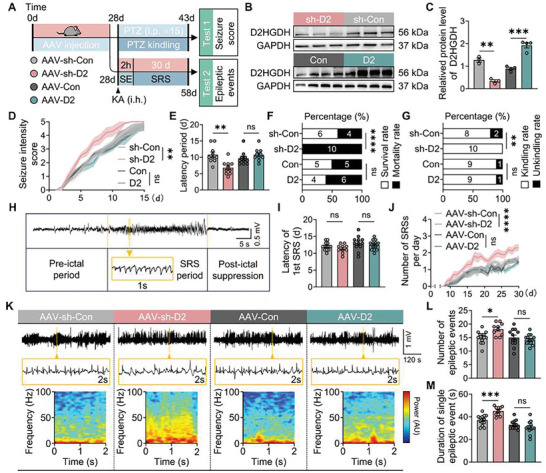
*D2HGDH* modulates seizure susceptibility and severity. A) Timeline for the PTZ kindling model in mice and the KA‐induced epilepsy model, comparing four AAV‐hysn groups (AAV‐Sh‐Con vs AAV‐Sh‐*D2HGDH* and AAV‐Con vs AAV‐*D2HGDH*). B) Representative images from a western blot analysis of the four groups to assess the efficiency of knocking down or overexpressing the *D2HGDH* gene. C) Quantified data for *D2HGDH* protein levels following infection with AAV (AAV‐Sh‐Con vs AAV‐Sh‐*D2HGDH*, *n* = 3 per group; AAV‐Con vs AAV‐*D2HGDH*, *n* = 4 per group), analyzed using Student's *t*‐test. D) Quantified data for seizure intensity score in PTZ kindling model mice across the four AAV‐hysn groups, analyzed by two‐way ANOVA; *n* = 10 per group. E) Quantified data for latency periodin PTZ kindling model mice with the four AAV‐hysn groups, analyzed by one‐way ANOVA; *n* = 10 per group. F) Survival rate and mortality of PTZ kindling model mice across the four AAV‐hysn groups, analyzed using Fisher's exact test. G) Kindling rate of PTZ in the four AAV‐hysn groups, analyzed by Fisher's exact test. H) Representative images of SRSs; scale bar as indicated. I) Latency to the 1st SRS after SE, analyzed by one‐way ANOVA. J) Number of SRSs per day following SE, analyzed by two‐way ANOVA. K) Representative images of LFPs in hippocampal regions of KA‐induced epilepsy mice; scale bar as indicated. L) Number of epileptic events, analyzed by one‐way ANOVA. M) Duration of single epileptic event, analyzed by one‐way ANOVA (AAV‐Sh‐Con, *n* = 13 vs AAV‐Sh‐*D2HGDH*, *n* = 10; AAV‐Con, *n* = 12 vs AAV‐*D2HGDH*, *n* = 13).

To investigate the potential role of *D2HGDH* in epilepsy, an adeno‐associated virus (AAV) was injected into the hippocampal region to silence or overexpress this gene in naïve C57BL/6J mice (**Figure** **2**A). The efficiency of the viral intervention was confirmed using western blot analysis in mice (Figure 2B,C).

In addition to genetic modification, two primary methodologies for modeling epilepsy in animals are commonly employed:^[^ ^17^ ^]^ 1) a kindling model, which use a chemical agent such as PTZ, and 2) a chronic epilepsy model, which utilize KA to induce spontaneous recurrent seizures (SRSs) following the structural damage caused by sustained status epilepticus (SE). First, a PTZ kindling model was established to assess the impact of acute seizures using Racine scale, kindling rate, and post‐injection mortality as indicators.^[^ ^18^ ^]^ *D2HGDH* knockdown resulted in an increase in these indicators (Figure 2D,F,G), with the exception of latency(Figure 2E), while overexpression did not demonstrate any significant differences (Figure 2D–G). Second, a KA‐induced epilepsy model^[^ ^19^ ^]^ was established to investigate the effects of upregulation or downregulation of *D2HGDH* on the chronic phase of seizures in KA mice using video monitoring and local field potentials (LFPs) recording^[^ ^20^ ^]^ (Figure 2H). *D2HGDH* knockdown increased both the number (Figure 2L) and duration (Figure 2M) of epileptic events in LFPs, as well as the number of SRSs in seizure behavior monitoring (Figure 2J), but did not affect SRSs latency (Figure 2I), compared to that in controls. In contrast, no significant differences were observed after *D2HGDH* overexpression.

Previous research has shown that spectral analysis of LFPs power can accurately measure the degree of hippocampal neuronal hyperexcitation during seizures in KA‐induced epilepsy models.^[^ ^21^ ^]^ Moreover, a direct relationship exists between these two variables. In this study, we analyzed the LFPs power within the frequency range of 0–100 Hz in the four experimental groups. Notably, downregulation of *D2HGDH* seemed to increase the LFPs power within the same frequency band (Figure 2K). Collectively, these findings suggest that downregulation of *D2HGDH* may modulate seizure susceptibility and severity, whereas upregulation of *D2HGDH* has no significant effect.

### 2.3 Effects of Upregulation and Downregulation of *D2HGDH* on Neuronal Function in the Hippocampus

To investigate the potential regulation of hippocampal neuronal function by *D2HGDH* gene expression, we conducted electrophysiological and morphological analyses of hippocampal neurons in C57BL/6J mice following the upregulation and downregulation of *D2HGDH*.

**Figure 3:**
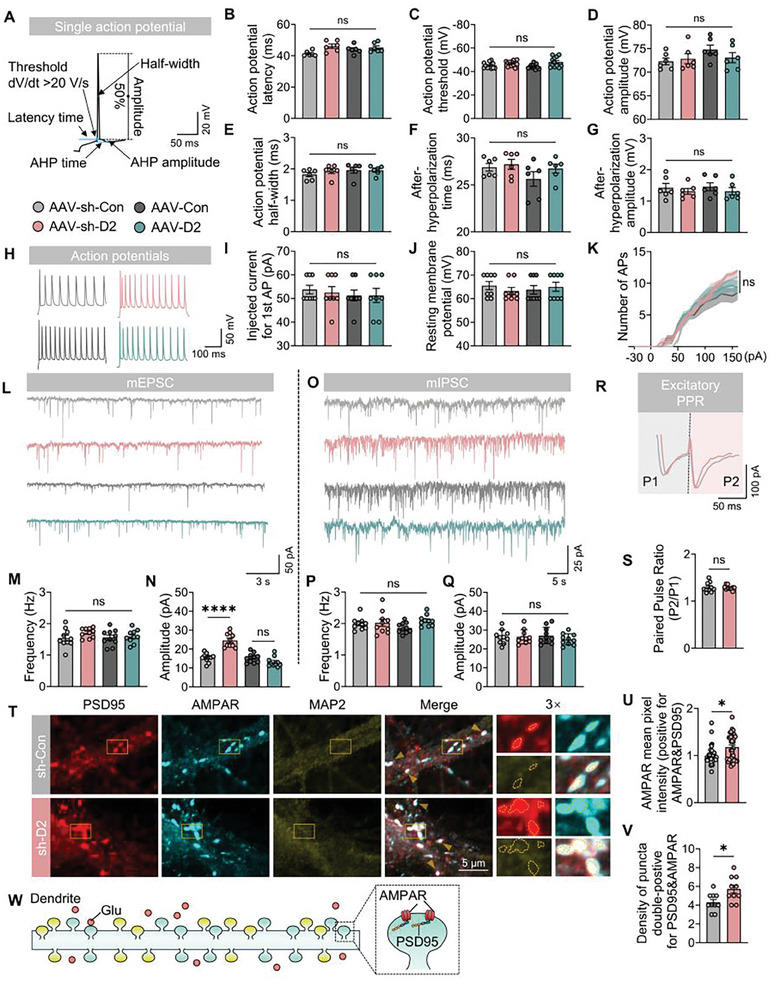
Effects of upregulation and downregulation of *D2HGDH* on neuronal electrophysiological function. A) A representative single action potential illustrating the measurement of action potential parameters obtained from CA1 neurons of four AAV groups (AAV‐Sh‐Con vs AAV‐Sh‐*D2HGDH* and AAV‐Con vs AAV‐*D2HGDH*). The parameters include action potential latency, threshold, amplitude, half‐width, AHP time, and AHP amplitude. Scale bar as indicated. B–G) Quantified summary data for action potential latency n=6, action potential threshold n=12, action potential amplitude n=6, action potential half‐width n=6, AHP time n=6, and AHP amplitude n=6 were analyzed using the Kruskal–Wallis test. H) Representative images of action potential traces; scale bar as indicated. I) Quantified data for the injected current required for the first action potential, analyzed using the Kruskal–Wallis test; *n* = 8. J) Quantified data for resting membrane potential (absolute value), analyzed using the Kruskal–Wallis test; *n* = 8. K) Quantified data for the firing frequency of action potentials at different stimulation currents, analyzed using two‐way ANOVA; *n* = 8. L) Representative traces of mEPSCs in four AAV groups; scale bar as indicated. M) Quantified data for mEPSC frequency, analyzed using one‐way ANOVA; *n* = 10. N) Quantified data for mEPSC amplitude, analyzed using one‐way ANOVA; *n* = 10. O) Representative traces of mIPSCs in four AAV groups; scale bar as indicated. P) Quantified data for mIPSC frequency, analyzed using one‐way ANOVA; *n* = 10. Q) Quantified data for mIPSC amplitude, analyzed using one‐way ANOVA; *n* = 10. R) Representative images for EPSC‐PPR (P2/P1) in two AAV groups (AAV‐Sh‐Con vs AAV‐Sh‐*D2HGDH*); scale bar as indicated. S) Quantified data for EPSC‐PPR (P2/P1), analyzed using the Mann–Whitney test; *n* = 10. T) Co‐staining of PSD95, AMPAR, and MAP2 in C57BL/6J cultured neurons (AAV‐Sh‐Con vs AAV‐Sh‐*D2HGDH*); scale bar as indicated. U) Quantified data for the mean pixel intensity of AMPAR (positive for AMPAR and PSD95), analyzed using the Mann–Whitney test; *n* = 39. V) Quantified data for the density of puncta double‐positive for PSD95 and AMPAR per 20 µm, analyzed using Student's *t*‐test; *n* = 10. W) Schematic diagram of an excitatory postsynapse.

First, to investigate the potential influence of *D2HGDH* on the intrinsic excitability and firing characteristics of CA1 neurons, we conducted whole‐cell current‐clamp recordings across four distinct groups (**Figure** **3**A). No significant differences were observed in the action potential latency (Figure 3B), threshold (Figure 3C), amplitude (Figure 3D), half‐width (Figure 3E), afterhyperpolarization (AHP) time (Figure 3F), or AHP amplitude (Figure 3G), resting membrane potential (Figure 3J), or first action potential injection current (Figure 3I) evoked by the substrate current (Figure 3H). Furthermore, trains of action potential elicited by current injections at different levels showed similar firing frequencies in four AAV‐hysn groups (Figure 3K). These results indicated that *D2HGDH* does not promote epileptic responses by altering intrinsic neuronal excitability. Second, we used a whole‐cell voltage‐clamp recordings to investigate whether *D2HGDH* affects synaptic electrical activity (Figure 3L,O). We observed no changes in the frequency of miniature excitatory postsynaptic currents (mEPSCs) (Figure 3M), but an increase in the amplitude of mEPSCs was noted after *D2HGDH* knockdown (Figure 3N). The frequency and amplitude of the miniature inhibitory postsynaptic currents (mIPSCs) remained unchanged (Figure 3P,Q), suggesting that changes in *D2HGDH* knockdown might impair glutamatergic synaptic transmission without affecting GABAergic synaptic transmission.

In general, changes in mEPSC amplitude indicate an increase in the ligand‐receptor binding capacity at excitatory synapses. The increase in the amplitude of the mEPSCs may be due to an increased release of presynaptic membrane transmitters, an increase in the total number of postsynaptic membrane receptors, or a combination of both. Here, downregulation of *D2HGDH* did not appear to change the frequency of mEPSCs but significantly increased the amplitude, implying that the only change was most likely in the receiving receptor factors. To further determine whether the cause of the change in the probability of presynaptic glutamate release is involved, we investigated the EPSC‐mediated paired‐pulse ratio (PPR) in hippocampal synapses (P2/P1) but found no significant differences between the two groups. (Figure 3R,S).

In addition, we investigated the morphology of hippocampal neurons in C57BL/6J mice after specific upregulation or downregulation of AAV‐hysn (Figure S2A–D, Supporting Information). Most excitatory synapses in the mature mammalian brain are located on spines, which are categorized by their shape as filopodium, thin, stubby, mushroom‐shaped, or cup‐shaped^[^ ^22^ ^]^ (Figure S2E, Supporting Information). Typically, the spine maintains optimal levels of synaptic activity, and alterations in spine density correlate with changes in neurotransmission efficacy.^[^ ^23^ ^]^ Furthermore, glutamate receptor activity significantly influences the morphology of spines.^[^ ^24^ ^]^ No effect was observed on the number of functional synapses in C57BL/6J mice; however, downregulation of *D2HGDH* increased the proportion of immature dendritic spines in neurons in the CA1 (Figure S2F, Supporting Information), CA3 (Figure S2G, Supporting Information), and DG regions (Figure S2H, Supporting Information), as well as the density of dendritic spines (Figure S2L, Supporting Information). No significant difference in spine length was observed in the CA3 and DG regions of the hippocampus (Figure S2J,K, Supporting Information), with the exception of the CA1 region (Figure S2I, Supporting Information).

Accumulated morphological and electrophysiological data suggested that *D2HGDH* modulates the postsynaptic efficacy of excitatory hippocampal neurons. Finally, our immunofluorescence co‐localization staining (MAP2&PSD95&α‐amino‐3‐hydroxy‐5‐methyl‐4‐isoxazolepropionic acid receptor (AMPAR), Figure 3T) on cultured primary neurons indicated that the density of dendritic spines was increased (Figure 3V) and mean AMPAR fluorescence intensity elevated (Figure 3U) with PSD95 and AMPAR co‐localizing in AAV‐Sh‐*D2HGDH*, compared with the AAV‐Sh‐con group However, the mean vesicular glutamate transporter (VGLUT) fluorescence intensity was not significantly different between the two groups (Figure S2M,N, Supporting Information). In conclusion, postsynaptic excitatory receptors are modulated by *D2HGDH*, which may explain the variation observed in our animal models.

### 2.4 Glutathione Reduced (GSH)/Glutathione Oxidized (GSSG) Ratio Imbalance and ROS Changes after *D2HGDH* Downregulation

**Figure 4:**
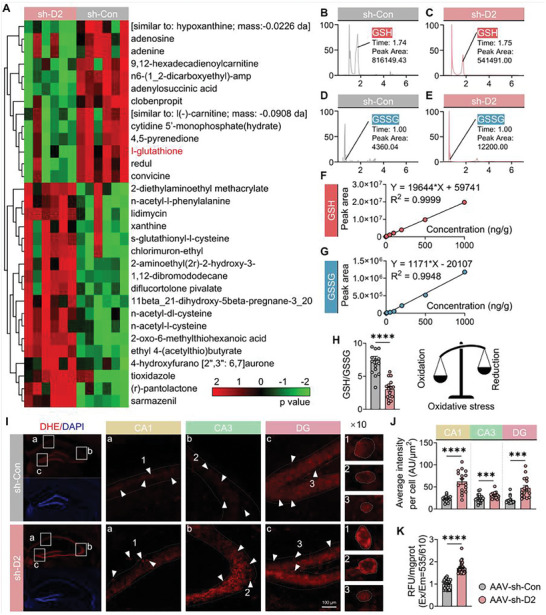
GSH/GSSG imbalance and ROS changes after downregulation of *D2HGDH* in mice using AAV. A) Heat map of changes in differential metabolite levels between AAV‐Sh‐*D2HGDH* (*n* = 6) and AAV‐Sh‐con (*n* = 6) groups. B–E) HPLC results display chromatograms and peak areas of GSH in the AAV‐Sh‐con (B, *n* = 16) and AAV‐Sh‐*D2HGDH* (C, *n* = 16) groups, as well as GSSG in the AAV‐Sh‐con (D, *n* = 16) and AAV‐Sh‐*D2HGDH* (E, *n* = 16) groups. F,G) Linear regression analysis conducted using GraphPad Prism 9.0 yielded the following equations: *Y* = 19644*X + 59741 with *R* ^2^ = 0.9999 for GSH (F); and *Y* = 1171*X − 20107 with *R* ^2^ = 0.9948 for GSSG (G). H) The redox status of glutathione was evaluated based on the ratio of GSH to GSSG for the results presented in parts (B)–(E), corrected using the standard curves provided in (F) and (G), and analyzed with Student's *t*‐test (AAV‐Sh‐Con vs AAV‐Sh‐*D2HGDH*, *n* = 16). I) ROS levels in the AAV‐Sh‐Con and AAV‐Sh‐*D2HGDH* groups were assessed using DHE, including the CA1, CA3, and DG regions; scale bar as indicated. J) Quantified data for average intensity per cell (AU/µm^2^) of fluorescence quantification of ROS in the hippocampus CA1, CA3, and DG regions, respectively, were analyzed using the Mann–Whitney test; *n* = 16 per group. K) Quantified data of fluorescence quantification of ROS measured by microplate reader in the entire hippocampus, normalized against the AAV‐Sh‐Con group, were analyzed using Student's *t*‐test; *n* = 16 per group.

To explore the molecular mechanism by which *D2HGDH* regulates seizures, mass‐spectrometry‐based untargeted metabolomics was used to examine metabolic changes after *D2HGDH* intervention (**Figure** **4**A). Notably, the GSH content was significantly lower in the *D2HGDH* downregulation group than in the control group. In biological systems, moderate oxidative conditions can lead to the conversion of GSH to its oxidized form, GSSG, through interactions with protein sulfhydryl groups. Normally, GSH predominates, with GSSG present at much lower levels.^[^ ^25^ ^]^ Research has shown that GSH makes up over 90% of the total soluble glutathione (GSH + 2GSSG).^[^ ^26^ ^]^ Elevated GSSG levels and reduced GSH/GSSG ratios are indicative of increased oxidative stress in vivo.^[^ ^27^ ^]^ First, we quantified the levels of GSH and GSSG in the hippocampus of the *D2HGDH* downregulation group (AAV‐Sh‐*D2HGDH*) and the control group (AAV‐Sh‐con) using high‐performance liquid chromatography (HPLC) (Figure 4B–E). The GSH/GSSG ratio, which was determined after adjusting for linear regression (Figure 4F,G; Table S2, Supporting Information), showed a significant decrease in the *D2HGDH*‐knockdown group, indicating enhanced conversion of GSH to GSSG (Figure 4H). Differential metabolite analysis demonstrated a clear distinction between the *D2HGDH* downregulation group and the control group (Figure S3A,B, Supporting Information). Kyoto Encyclopedia of Genes and Genomes (KEGG) pathway analysis showed that the differential metabolites (AAV‐Sh‐*D2HGDH* vs AAV‐Sh‐con) were enriched in common pathways, such as glutathione metabolism and ROS metabolism (Figure S3C and Table S3, Supporting Information). GSH is an essential endogenous small‐molecule antioxidant that protects the body against oxidative stress‐induced damage by binding to ROS for redox reactions.^[^ ^28^ ^]^ Second, we performed dihydroethidium (DHE) staining on C57BL/6J mouse hippocampus to assess ROS levels. The fluorescence in the hippocampus, including the CA1, CA3, and DG areas (Figure 4I), exhibited a significant difference (Figure 4J) in the AAV‐Sh‐*D2HGDH* group compared to that in the AAV‐Sh‐con group. Furthermore, the total ROS level in the entire hippocampus was also elevated and normalized to that of the control (Figure 4K). The findings presented above indicate that downregulation of *D2HGDH* increased seizure activity and oxidative stress (owing to the depletion of GSH, an important antioxidant, and excessive accumulation of ROS), suggesting that *D2HGDH* may affect the pathophysiology of epilepsy through ROS production in vivo.

### 2.5 Peroxiredoxin 6 is a Potential Factor Affecting ROS Generation

**Figure 5:**
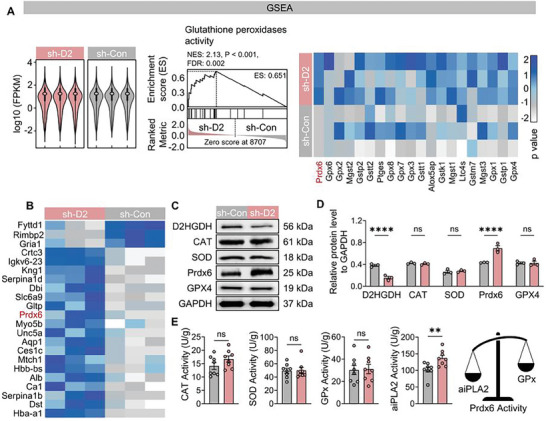
Prdx6 is a potential factor affecting ROS generation in mice using AAV. A) FPKM (fragments per kilobase million) values from transcriptome analysis indicate the expression of genes in the AAV‐Sh‐*D2HGDH* and AAV‐Sh‐Con groups (*n* = 3 per group). Gene sets induced by Gpxs activity were identified through GSEA in the AAV‐Sh‐*D2HGDH* and AAV‐Sh‐Con groups (NES, normalized enrichment score). A heat map illustrates the log10‐fold expression levels of Gpxs activity‐induced gene sets (*n* = 3 per group). B) A heat map displays the log10‐fold expression levels of differentially abundant proteins identified through TMT‐based quantitative proteomics in the AAV‐Sh‐*D2HGDH* and AAV‐Sh‐Con groups (*n* = 3 per group). C) Representative images show the levels of proteins associated with oxidative stress (*D2HGDH*, CAT, SOD, Prdx6, GPX4, and GAPDH) in the AAV‐Sh‐*D2HGDH* and AAV‐Sh‐Con groups. D) Quantified data for the levels of proteins associated with oxidative stress (*D2HGDH*, CAT, SOD, Prdx6, GPX4), normalized to GAPDH, were analyzed using two‐way ANOVA (*n* = 3 per group). E) Quantified data for the activity of proteases associated with oxidative stress (CAT, SOD, GPx, and aiPLA2) were analyzed using Student's *t*‐test in the AAV‐Sh‐*D2HGDH* and AAV‐Sh‐Con groups (*n* = 8 per group), demonstrating the superiority of aiPLA2 activity over that of GPx.

Given the impact of *D2HGDH* knockdown on glutathione metabolism, we initially explored the potentially affected genes using GSEA (**Figure** **5**A). Subsequently, we conducted a quantitative protein comparative analysis between the two groups (AAV‐Sh‐*D2HGDH* vs AAV‐Sh‐con) using tandem mass spectrometry tag (TMT)‐based quantitative proteomics (Figure 5B). Notably, the redox protein peroxiredoxin 6 (Prdx6) was upregulated in both analyses (GSEA and TMT). Prdx6 is a bifunctional enzyme with glutathione peroxidases (GPxs) and acid calcium‐independent phospholipase A2 (aiPLA2) activities.^[^ ^29^ ^]^ It is part of the antioxidant system, along with superoxide dismutase (SOD), catalase (CAT), and GPx, which protect cells by neutralizing superoxide radicals and hydrogen peroxide.^[^ ^30^ ^]^ SOD turns superoxide into hydrogen peroxide, which can be converted into hydroxyl radicals via the Fenton reaction.^[^ ^31^ ^]^ GPx and Prdx6 reduce hydrogen peroxide to water and GSSG using GSH. CAT plays a crucial role in enzymatic defense by neutralizing H_2_O_2_ to produce water (Figure S4D, Supporting Information). However, whether ROS regulation by *D2HGDH* is accompanied by changes in the expression of these proteins remains unclear.

Our findings indicate that *D2HGDH* knockdown resulted in a significant increase in the expression of Prdx6, while the levels of SOD, CAT, and glutathione peroxidase 4 (GPX4) remained unchanged in the hippocampal brain tissue of epileptic patients (Figure 5C,D), and activity assays showed that increased levels of Prdx6 resulted in its aiPLA2 activity being superior to its GPx activity (Figure 5E). The findings suggest that Prdx6 may function as a potential downstream protein of *D2HGDH*, contributing to the modulation of the epileptic phenotype. Research on the mechanism by which oxidative stress regulates epilepsy has long focused on glial cells,^[^ ^32^ ^]^ while the role of neurons has been relatively neglected. In this study, we investigated the potential relationship between synaptic function and seizure mechanisms by targeting AAV‐hysn to interfere with neuronal *D2HGDH* expression in the C57BL/6J hippocampus.

Our bioinformatics analysis strongly suggested that alterations in intracellular metabolism regulated by AAV‐Sh‐*D2HGDH* affected the synaptic function of neurons (Figure S4A and Table S4, Supporting Information), including changes in the glutamatergic synapse pathway, which were consistent with those identified by our electrophysiological experiments. In addition, to determine the correlations between the levels of various metabolites (Figure S4B, see Table S5.1, Supporting Information for details) and proteins (Figure S4B, see Table S5.2, Supporting Information for details), we conducted an UpSet combined analysis of differentially enriched metabolites and proteins in the two comparison groups (Figure S4C and Table S6, Supporting Information). Downregulation of *D2HGDH* affected pathways related to glutathione metabolism, influencing the ROS regulatory system and compromising cellular receptor–ligand binding.

Previous research has shown that disruption of redox homeostasis in the brain is the basis of synaptic transmission and plasticity impairment and that postsynaptic receptors are an important part of the synaptic transmission structure.^[^ ^33^ ^]^ Moreover, AMPAR activity has been shown to be regulated by the redox state in vivo, suggesting that ROS increase AMPAR electrophysiological activity.^[^ ^34^ ^]^ Our electrophysiological investigation revealed that downregulation of *D2HGDH* may increase the number of AMPARs without altering their intrinsic excitability. The results of these previous studies, together with those of the present study, suggest that the regulation of the number of AMPARs may be related to the expression of synaptic proteins in neurons; however, this requires further investigation. Taken together, these results indicate that downregulation of *D2HGDH* may regulate GSH/Prdx6/ROS metabolism, thereby linking the intracellular glutathione redox reaction (mechanism), neuronal synaptic function (phenotype), and seizures (disease) and elucidating the potential effects of individual genes on epilepsy.

### 2.6 aiPLA2 Inhibitor MJ33 Antagonizes the Increased Susceptibility to Epilepsy and Oxidative Stress Levels Caused by *D2HGDH* Knockdown

**Figure 6:**
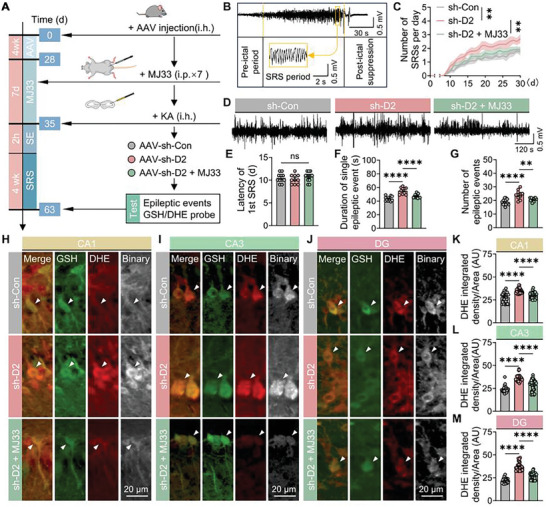
aiPLA2 inhibitor MJ33 antagonizes the increased susceptibility to epilepsy and ROS levels caused by *D2HGDH* knockdown. A) Timeline and experimental procedure in a KA‐induced epilepsy model infected with AAV‐hysn and MJ33. The animals were divided into three groups: AAV‐Sh‐Con, AAV‐Sh‐*D2HGDH*, and AAV‐Sh‐*D2HGDH*+MJ33. B) Representative images of SRSs; scale bar as indicated. C) The number of SRSs per day following SE was analyzed using two‐way ANOVA, *n* = 10. D) Representative images of epileptic events; scale bar as indicated. E) Quantified data for the latency to the 1st SRS, analyzed using one‐way ANOVA, *n* = 10. F) Quantified data for the duration of single epileptic event, analyzed using one‐way ANOVA. G) Quantified data for the number of epileptic events, analyzed using one‐way ANOVA (AAV‐Sh‐Con, *n* = 14; AAV‐Sh‐*D2HGDH*, *n* = 11; AAV‐Sh‐*D2HGDH*+MJ33, *n* = 13). H–J) GSH (green) and DHE (red) probes tracking hippocampal neurons in the CA1, CA3, and DG regions; scale bar as indicated. K–M) ROS content in the hippocampus CA1, CA3, and DG, respectively, quantified using DHE and analyzed by the Kruskal–Wallis test; *n* = 37 per group.

Accumulating evidence suggests that an increase in oxidative stress resulting from *D2HGDH* knockdown is associated with changes in Prdx6 enzyme activity. Initially, we selected the classical aiPLA2 inhibitor MJ33 for the KA‐induced epilepsy model involving *D2HGDH* knockdown.^[^ ^29^, ^35^ ^]^ Consecutive daily intraperitoneal injections of MJ33 were administered for 7 d before KA injection (**Figure** **6**A). We observed partial improvement in epileptic events (Figure 6D), specifically a reduced number and duration of epileptic events after MJ33 treatment (Figure 6F,G), as well as the number of SRSs (Figure 6C), but not the latency of the 1st SRS (Figure 6E).

Next, we used dual fluorescent probes to measure the immunofluorescence intensity of GSH and ROS levels before and after MJ33 intervention (Figure 6H–J). Notably, the dominant role of aiPLA2 activity of Prdx6 in ROS production was further confirmed by the finding that MJ33 significantly reduced ROS levels in the hippocampus in vivo (Figure 6K–M) Similarly, *D2HGDH* knockdown reduced GSH (Figure S5C, Supporting Information) but increased ROS levels (Figure S5D, Supporting Information) in cultured C57BL/6J neurons (Figure S5B, Supporting Information). However, this effect was reversed by treatment with the aiPLA2 inhibitor MJ33 (Figure S5C,D, Supporting Information). Neuronal activity was measured using the Cell Counting Kit‐8 (CCK8) assay without changes (Figure S5E, Supporting Information). This indicated that MJ33 reversed the negative impact of *D2HGDH* knockdown on neuronal oxidative stress levels without affecting neuronal viability. Taken together, the in vivo and in vitro results suggest that the aiPLA2 activity of Prdx6 is a potential target of *D2HGDH* in regulating neuronal ROS production.

Furthermore, given that Prdx6 has distinct roles in different disease states, we performed immunofluorescence‐based co‐localization analysis of Prdx6 and various organelles (Figure S5F, Supporting Information). Fluorescence co‐localization analysis determines the spatial relationship between two molecules, providing visual, quantitative, and statistical assessments.^[^ ^36^ ^]^ We utilized Pearson's correlation coefficient (PCC) and Mander's co‐localization coefficient (MCC) to measure the spatial correlation and overlap between Prdx6 and organelles (Figure S5I–M, Supporting Information).Co‐localization was considered to exist when the PCC or MCC values were ≥0.5.^[^ ^37^ ^]^ Research has shown that Prdx6 is widely expressed in the cytoplasm under physiological conditions;^[^ ^38^ ^]^ however, its localization under pathological conditions remains controversial. Here, we found that Prdx6 was mainly localized to lysosomes and not mitochondria, which may be the basis for its ability to exert aiPLA2 instead of GPx activity (Figure S5G,H, Supporting Information).

## 3 Discussion

The *D2HGDH* gene encodes the enzyme *D2HGDH*, a mitochondrial enzyme classified within the FAD‐binding oxidoreductase/transferase 4 family. This enzyme is active in the heart, liver, and brain, where it facilitates the conversion of D2‐HG to 2‐ketoglutarate.^[^ ^39^ ^]^ In recent years, knowledge in this field has advanced significantly, largely due to progress in genomics. Research has demonstrated that mutations in the *D2HGDH* gene are responsible for d‐2‐hydroxyglutaric aciduria.^[^ ^11^, ^40^ ^]^ If these enzymatic assays capture a relevant cellular event, then the NADP/NADPH ratio and, consequently, the abundance of ROS may also be influenced by *D2HGDH* expression and mutational status.^[^ ^10b^ ^]^ This suggests that *D2HGDH* may be involved in the pathophysiology of epilepsy.^[^ ^15^ ^]^ However, previous studies have primarily focused on the metabolite, D2‐HG,^[^ ^10b^ ^]^ overlooking its role in the nervous system. In this study, we identified a novel role of *D2HGDH* in epilepsy regarding the potential regulation of synaptic oxidative damage in neurons. Interestingly, downregulation of *D2HGDH* appears to influence both the susceptibility and severity of epilepsy, whereas upregulation seems to have no significant effect. These results may be attributed to a gene dose effect, as observed in clinical cases of hydroxyglutarate metabolism epilepsy associated with *D2HGDH* loss.^[^ ^41^ ^]^ Alternatively, compensatory mechanisms may be involved; specifically, in instances of gene knockdown, intracellular compensatory processes that would otherwise be inactive or suppressed may be activated in response to gene overexpression.

In recent years, several non‐GPx‐dependent ROS regulation modes have been reported, including mechanisms involving DHODH, which inhibits ferroptosis in a GPx‐independent manner.^[^ ^42^ ^]^ For example, in ferroptosis cells, Prdx may sense lipid peroxides, leading to specific hyperoxidation of Prdx3.^[^ ^43^ ^]^ This superoxide stimulates ferroptosis by inhibiting cysteine uptake. In the presence of glutathione, GPX4 effectively scavenges lipid peroxides, thereby preventing oxidative stress. When GSH is depleted, another system may come into play; aiPLA2 is activated, leading to accumulation of peroxides that cannot be counteracted by other antioxidant mechanisms in vivo.^[^ ^44^ ^]^ Prdx6 has two opposing functions associated with its two active sites^[^ ^45^ ^]^ and also plays a role in regulating phospholipid conversion and preventing oxidative damage.^[^ ^29c^ ^]^ In most cases, Prdx6 functions as a GPx, using glutathione and metabolic peroxide ions as substrates for detoxification, thereby protecting the body from oxidative stress damage.^[^ ^46^ ^]^ Under certain pathological conditions, Prdx6 acts as a aiPLA2, causing cell membrane damage, inducing apoptosis,^[^ ^45^, ^47^ ^]^ and increasing ROS production by activating NADPH oxidase.^[^ ^46^ ^]^ The aiPLA2 activity of Prdx6 has been associated with the pathogenesis of Parkinson's disease and has been shown to hinder neurogenesis in the context of neurodegenerative disorders in transgenic mouse models.^[^ ^47^, ^48^ ^]^ Overexpression of Prdx6 has been demonstrated to accelerate the progression of Parkinson's disease, Alzheimer's disease, and experimental autoimmune encephalomyelitis in various animal models.^[^ ^48^, ^49^ ^]^ Therefore, Prdx6 activity may be relatively high in neurons.^[^ ^38^ ^]^ In addition, Prdx6 preferentially utilizes GSH as a reducing agent, indicating that low GSH levels may restrict Prdx6 activity.^[^ ^50^ ^]^ In this study, we found that a severe reduction in GSH content led to the predominance of aiPLA2 activity of Prdx6, resulting in ROS overproduction and decreased cellular antioxidant capacity.

Notably, our transcriptic KEGG enrichment analysis indicated that downregulation of *D2HGDH* may alter the expression of synapse‐related genes as follows: 1) glutamate receptor protein Gria1; 2) Shank2, a member of the Shank protein family that serves as a molecular scaffold for the postsynaptic density of excitatory synapses.^[^ ^51^ ^]^ In addition, TMT differential protein analysis indicated a variation in the content of Rimbp2 protein. It is believed that Rimbp2 plays an active role within the cytoplasmic component of the observed active zone.^[^ ^52^ ^]^ Changes in the expression of these proteins suggest that *D2HGDH* is involved in synaptic activity. The propagation of action potentials and the functioning of synapses are fundamental processes underlying neuronal activity. Neurons exhibit vigorous redox‐dependent metabolic activity, which is associated with increased production of ROS.^[^ ^53^ ^]^ Various antioxidant defense mechanisms operate to neutralize ROS, regulate redox‐dependent signaling pathways, and mitigate the harmful oxidative effects of ROS on cellular components.^[^ ^53a^ ^]^ An imbalance between the generation and neutralization of ROS results in oxidative stress, a condition characterized by the disruption of redox signaling and oxidative damage to biomolecules.^[^ ^54^ ^]^ Although neurons produce a large number of oxidative targets, their antioxidant defense systems are relatively weak, and they are susceptible to oxidative stress.^[^ ^55^ ^]^ The intracellular GSH content is ≈50% lower in neurons than in other cells.^[^ ^56^ ^]^ A novel and important finding of this study was that downregulation of *D2HGDH* altered the GSH/GSSG balance and ROS production in vivo. Our data demonstrate that Prdx6 is a potential target substrate of *D2HGDH* in epilepsy. Another important finding is that the aiPLA2 inhibitor MJ33 can effectively reduce ROS production and consequently alleviate seizures. In summary, our data suggest that *D2HGDH* may act as a regulator in the modulation of ROS pathways and that relatively low cytoplasmic GSH levels may limit GPx activity, leading to false‐positive feedback (accumulation of excess ROS via aiPLA2 activity) caused by increased Prdx6 expression.

As a sentinel to synaptic activity, ROS may directly enhance the synaptic function of excitatory neurons, potentially exacerbating seizures in mice.^[^ ^34^, ^44^ ^]^ From a metabolic perspective, the complex nature of neuronal redox homeostasis may explain the limited effectiveness of nutritional antioxidants in treating neurodegenerative disorders.^[^ ^57^ ^]^ However, concerns exist regarding the long‐term use of antioxidants as antiepileptic agents, which may be harmful rather than protective.^[^ ^33^ ^]^ Approximately half of the AMPAR are mobile within the synapse rather than aggregated.^[^ ^58^ ^]^ This suggests that ROS signaling may serve as a selective mechanism linking cellular metabolism to excitatory neurotransmission through either direct or indirect modulation of AMPAR function, ultimately leading to acute synaptic plasticity without affecting glutamate release from presynaptic terminals. Excessive *D2HGDH* knockdown, which influences ROS levels, resulted in an increase in AMPARs, which disrupted glutamate‐induced signaling. Moreover, increased extracellular oxidation enhances the potency of AMPAR‐induced active responses, specifically tension currents, at low concentrations.^[^ ^59^ ^]^ These observations help elucidate why the application of endogenous intracellular antioxidants such as glutathione is ineffective in patients with epilepsy.^[^ ^60^ ^]^ Prdx6 inhibition of aiPLA2 activity may represent a promising therapeutic strategy to mitigate neuronal oxidative stress and reduce seizures in patients with clinically refractory metabolic hydroxyglutarate epilepsy caused by *D2HGDH* deficiency.

However, our study has some limitations, because the effects of ROS on postsynaptic receptors were predominantly concentrated on AMPARs, and the potential involvement of NMDA receptors should not be overlooked.

## 4 Conclusion

This study demonstrated the localization of *D2HGDH* in TLE and C57BL/6J mouse brain tissue. Compared to brain tissues from individuals with TBI, *D2HGDH* content was increased and was mainly localized in neurons in tissues from TLE patients. The hippocampal excitatory neurons in mice abundantly express *D2HGDH*. Second, we explored the potential role of *D2HGDH* in epilepsy models (KA and PTZ models). *D2HGDH* knockdown aggravated epileptic events in a KA‐induced epilepsy model and increased the Racine score in a PTZ kindling model, whereas overexpression had no effect. Third, we investigated the functional effects of *D2HGDH* on C57BL/6J hippocampal neurons and found that downregulation of *D2HGDH* regulated AMPAR function and the proportion of mature dendritic spines in neurons. Finally, we explored the potential downstream pathways using *D2HGDH* knockdown together with metabolomics, proteomics, and GSEA. Our results suggest that the *D2HGDH*/GSH/Prdx6/ROS axis may be involved in the development of epilepsy and that MJ33, an inhibitor of aiPLA2, could potentially reverse this phenotype. In conclusion, targeting *D2HGDH* downregulation in C57BL/6J mice and neurons contributes to our understanding of the pathophysiology of epilepsy.

## 5 Experimental Section

### Animals and Human Brain Tissue—*Animals*

The male C57BL/6J mice utilized in this study were sourced from the Animal Experimentation Center at Chongqing Medical University and were housed under regulated environmental conditions (temperature: 22–24 °C; relative humidity: 50 ± 1%; 12‐h light/dark cycle, with illumination commencing at 08:00 h), with continuous access to standard food and water. This research received ethical approval from the Ethics Committee of the First Affiliated Hospital of Chongqing Medical University (2020‐821). At the commencement of the experiment, the mice were 28 days old and exhibited an average weight of ≈22–24 g. All procedures involving animals were conducted in compliance with the ARRIVE guidelines and adhered to the U.K. Animals (Scientific Procedures) Act of 1986, as well as the pertinent regulations established in the EU Directive 2010/63/EU concerning animal research.

### Human Brain Tissue

Postoperative brain tissues were obtained from patients diagnosed with TLE as well as from individuals who underwent temporal lobe resection during decompression surgery for TBI. This investigation employed clinical samples, specifically temporal lobe tissues from three TLE patients and three TBI patients without epilepsy (Table S1, Supporting Information for further details). The experimental procedures adhered to the principles outlined in the Declaration of Helsinki and were sanctioned by the hospital's Ethics Committee.

### Mouse Primary Neuron Culture

Hippocampal neurons were isolated from C57BL/6 lactating mouse pups within 24 h postnatally through the processes of trypsinization and centrifugation. The neurons were subsequently suspended in Dulbecco's Modified Eagle Medium supplemented with 10% fetal bovine serum and 1% antibiotics (penicillin/streptomycin). Following a 4‐h incubation period in a cell culture incubator at 37 °C (designated as IVD 0), the culture medium was replaced with a complete medium containing B27, l‐glutamine, 100 U mL^−1^ penicillin, and 100 µg mL^−1^ streptomycin (all sourced from Invitrogen), and the cultures were maintained at 37 °C with 5% CO_2_. The culture medium was refreshed by replacing half of it every 2–3 d. C57BL/6J primary neurons were cultured for 14 days to facilitate immunofluorescence analysis. The expression of *D2HGDH* gene was induced at IVD 0 by viral infection (1 µL per well).; subsequently, MJ33 (Sigma‐Aldrich, St. Louis, MO) was incorporated into the culture medium for immunofluorescence staining, utilization of immunofluorescence probes, CCK8 assays, and assessment of enzyme activity. For detailed information regarding the viral vectors, antibodies, and reagents used, please refer to Table S7 (Supporting Information).

### Method Details—AAV Vectors Construction and Injections—AAV Vectors Construction

The construction of four AAV9 vectors was completed, each carrying distinct elements: the full‐length mouse *D2HGDH* (labeled AAV‐*D2HGDH*), EGFP alone (labeled AAV‐con), *D2HGDH*‐RNAi (labeled AAV‐Sh‐*D2HGDH*), and a scrambled RNAi (labeled AAV‐Sh‐con). This work was conducted by OBiO Technology Co. (Shanghai, China). During the packaging of AAV‐*D2HGDH*, the vector pAAV‐hysn‐EGFP‐WPRE was utilized. Control vectors consisted of empty AAV constructs encoding EGFP. For the packaging of AAV‐Sh‐*D2HGDH*, the vector pAAV‐hysn‐EGFP‐3×FLAG‐miR30shRNA(NC)‐WPRE was used. The RNAi sequence targeting *D2HGDH* was designed as 5′‐GGACCTGGCTGCATTTGAATG‐3′, while the sequence for the scrambled RNAi, which does not target any mRNA in mice, was 5′‐CCTAAGGTTAAGTCGCCCTCG‐3′.

### AAV Vectors and Injections

Mice were randomly allocated into four groups according to the specific type of virus administered. To mitigate potential structural and functional changes in hippocampal neurons caused by estrogen, which may increase the risk of seizures, only male mice were used during the initial phase of the experiments. A total of 500 nL of viral particles (0.5–1 × 10^12^ units [TUs] mL^−1^) was delivered into the dorsal hippocampus using a glass microsyringe at a rate of 0.2 µL min^−1^ (anterior/posterior: −2.0 mm, medial/lateral: ±1.5 mm, dorsal/ventral: −1.5 mm). Following the injection, the pipette was held in place for an additional 5 min to ensure that the viral particles did not flow back through the injection probe. After a duration of over four weeks, which was necessary for the virus to exert its effects, EGFP indicated the effect of the viral intervention in the hippocampus. To assess the impact of *D2HGDH* knockdown or overexpression, western blotting was used.

### Drug Trials

MJ33 (experimental dose: 500 × 10^−9^ m kg^−1^) was intraperitoneally injected once daily for 7 d before KA injections (Figure S6A, Supporting Information). Male C57BL6/J mice aged 7 weeks were used for the MJ33 dose‐screening study (Figure S6B–E, Supporting Information). The mice were divided into groups receiving doses of 175500 and 1500 × 10^−9^ m kg⁻¹ or 125, 250, 500, and 750 × 10^−9^ m kg⁻¹ in each group based on the concentration of MJ33. Each group received an intraperitoneal injection of MJ33 for 7 d. The control mice were intraperitoneally injected with 10% DMSO and 90% saline. Male mice were anesthetized using isoflurane, administered at concentrations of 3%–5% for induction and 1%–2% for maintenance, and subsequently positioned within a stereotaxic apparatus. A total volume of 1.0 nmol of KA, dissolved in 50 nL of saline, was delivered into the right hippocampus over 3 min using a 0.5 µL microinjector. The coordinates for the injection were determined relative to bregma, specifically at anteroposterior (AP) ‐1.6 mm, mediolateral (ML) ‐1.5 mm, and dorsoventral (DV) ‐1.5 mm. The microinjector was held in place for an additional 5 min to minimize the likelihood of backflow. Furthermore, MJ33 was incorporated into the cell culture media at a final concentration of 20 × 10^−6^ m (according to the provided guidelines), administered 24 h before treatment (see Figure S5A, Supporting Information).

### PTZ Kindling Model and KA‐Induced Epilepsy Model

To establish a PTZ kindling model, a subthreshold dose of PTZ (35 mg kg^−1^) was injected intraperitoneally into mice in each group at regular intervals of 24 h every day for 15 d. Based on the observations, mice exhibit fewer seizures in the morning and more in the afternoon, with a noted decrease in excitement during the morning hours. In addition, all injections were scheduled between 09:00 and 12:00 in Beijing to eliminate the influence of confounding factors on seizure occurrence. After each injection of PTZ, the mice were observed for a minimum of 30 min. Seizures were evaluated using the Racine scale, which categorizes them as follows: Grade 0: indicates no alteration in behavior; Grade 1: is characterized by a sudden cessation of activity, accompanied by motionless staring and potentially orofacial automatism; Grade 2: involves head nodding; Grade 3: is defined by forelimb clonus in conjunction with a lordotic posture; Grade 4: includes forelimb clonus accompanied by rearing and falling; and Grade 5: represents generalized tonic‐clonic seizures, which result in a loss of postural tone and frequently lead to erratic jumping and, in some cases, death (see Video S1, Supporting Information). Only mice with at least three consecutive seizures scoring 4 or 5 were considered fully kindled.

To develop a KA‐induced epilepsy model, a single hippocampus injection of KA (1.0 nmol in 50 nL of saline) was administered to animals in each group to model TLE, followed by close monitoring for 2 h after KA injections. Upon detection of persistent SE seizures, the mice were immediately sedated via an intraperitoneal injection of diazepam. Mice that exhibited confirmed SE seizures and subsequently recovered from sedation were returned to their respective cages for further video monitoring. Mice treated with KA develop SRSs following brain injury caused by SE. SRSs typically emerged after the mice survived both the acute and latency periods. SRSs were evaluated on Racine scale, and only seizures with a score of 3 or higher were recorded to determine the number of SRSs and the latency period between SE termination and the 1st SRS. This marks the transition to what is termed a chronic epileptic model. Notably, owing to the mortality of some mice during the SE period following KA injections, the final number of mice included in the statistical analysis was less than 15 per group.

### LFPs Recording

Two ground stainless steel screws were implanted into the front of the skull using platinum‐iridium alloy microwires with a diameter of 25 µm. Plexus was transplanted to right side of the dorsal hippocampus (anterior/posterior, 1.6 mm; medial/lateral, 1.6 mm; dorsal/ventral, 1.5 mm). The catheter, microwire, and U‐shaped structure were then attached to the skull. The 2‐h LFPs recording were conducted five weeks after the SE induced by KA injection. The LFP recordings were filtered at 0.1–500 Hz, preamplified by a factor of 1000, digitized at a rate of 4 kHz, and recorded using a MAP data acquisition system (Plexon, Dallas, TX). Using a NeuroExplorer (Nex Technologies, Littleton, MA) to analyze LFPs recording data. Epileptic events are defined as a series of spontaneous episodic discharges that last for 5 s or longer, exhibit high‐amplitude peak activity at least 2 standard deviations above baseline, and have a frequency exceeding 1 Hz.

### Immunofluorescence

Mouse brain tissue samples were obtained under anesthesia and subsequently fixed in 4% paraformaldehyde for 24 h. Following fixation, the samples underwent a dehydration process using increasing concentrations of sucrose (15%, 20%, and 25%) and were then embedded in optimal cutting temperature compound before being frozen in isopentane at −80 °C. Coronal sections of the tissue were prepared at a thickness of 20–25 µm using a Leica CM1950 cryostat. The samples were then permeabilized in a solution containing 3% bovine serum albumin and 0.3% Triton X‐100 in PBS for 30 min at room temperature. The tissue slices were subsequently placed in a solution containing primary antibodies (*D2HGDH*, MAP2, GFAP, PSD95, GAD67; details in Table S7, Supporting Information) and incubated overnight at 4 °C. Following this incubation, the slices were washed twice with PBS for 10 minutes after each primary antibody. They were then incubated with the appropriate secondary antibodies (488, CY3, CY5; details in Table S7, Supporting Information) and Hoechst for 1 h at room temperature. Triple fluorescent staining (MAP2, GFAP, and *D2HGDH*) and double fluorescent staining (PSD95 or GAD67 costained with *D2HGDH*) were performed. Finally, the slices were washed twice with PBS (10 min per wash) and fixed on slides with coverslips and antifade solution. Images were captured using a laser scanning confocal microscope.

C57BL/6J hippocampal neurons cultured in vitro for 14 d were stained following the same general method: double fluorescent staining using LAMP1 or AIF co‐staining with Prdx6, triple fluorescent staining with MAP2, PSD95, and AMPAR, or MAP2, SYN, and VGLUT. Specifically, cultured hippocampal neurons labeled with 1 × 10^−6^ m of LiveReceptor AMPAR were fixed, permeabilized, and immunostained using anti‐PSD95 and anti‐MAP2 antibodies. The fluorescein signals corresponded well with the signal of anti‐PSD95 antibodies (details are in Table S7, Supporting Information).

### Image Analysis

To visualize immunofluorescent labeling, images were captured using an AxioImager (Carl Zeiss) equipped with an optical sectioning component and a digital camera (AxioCam; Carl Zeiss). Image acquisition was performed with AxioVision acquisition software (Carl Zeiss) in conjunction with a Nikon Eclipse Ni‐E microscope, which features a motorized XY stage to facilitate in situ imaging. After acquisition, the images were merged using NIS Elements software (Nikon). In some instances, linear adjustments to contrast and brightness were applied. Fiji's cell counting principle is based on particle analysis. Briefly, this study distinguished between the CA1, CA3, and DG regions of the hippocampus, and each DHE‐positive cell was labeled manually. Fiji software was used to quantify fluorescence intensity (binary) and area by setting a uniform threshold and calculating the average fluorescence for each area. GSH‐positive cells were included in the statistical standard for hippocampal slices or hippocampal neurons in vitro that were positive for the GSH/ROS dual fluorescent probes. Red indicates positive results for DHE, whereas green indicates GSH positivity, where AU is an arbitrary unit. Using NIH FIJI/ImageJ, raw images were analyzed to plot fluorescence intensity curves, which included distance (in pixels), mean fluorescence area, and integrated density, along with various adjacent background values. The corrected total cell fluorescence (CTCF) was calculated using the following equation: CTCF = integrated density (the area of the selected cell multiplied by the average fluorescence relative to the background). PCC and MCC were used as statistical measures of co‐localization to determine the degree of spatial correlation and the proportion of spatial overlap between the two proteins. Co‐localization was considered to exist when the PCC or MCC values were ≥0.5.

### Measurement of ROS and GSH Levels

ROS intensity was measured according to the DHE kit instructions (Biomart, Beijing, China, Cat. No. HR8821) and GSH intensity was measured according to the manufacturer's monochlorobimane (mBBr) instructions (MCE, New Jersey, USA, Cat. No. HY‐101899). For the whole hippocampal slices, fresh samples were incubated with serial dilutions for 1 h and then with 10 × 10^−3^ m of oxidation‐sensitive fluorescent probe DHE and mBBr in the dark for 30 min at 37 °C. For C57BL/6J primary neurons, mBBr was added to the plate at a final concentration of 1 × 10^−6^ m from a working solution of 1 × 10^−3^ m, while DHE was 1 × 10^−6^ m. Then, fluorescence intensity for DHE was subsequently measured using confocal microscopy; for proteins in vitro and in vivo, fluorescence intensity (RFU)/protein concentration (mg protein) (Ex/Em = 535/610) was used to represent ROS intensity and fluorescence intensity (RFU/protein concentration (mg protein) (Ex/Em = 405/526) was used to represent GSH intensity by microplate reader; the fluorescence intensity of the control sample in the experiment was used as the correction coefficient and the percentage of the fluorescence intensity value of the sample to be tested in the control sample was used to compare the intensity of ROS and GSH (protein concentration is independent).

### HPLC Analysis

The brains were removed from C57BL/6J mice, rinsed with ice‐cold PBS, and snap‐frozen in liquid nitrogen. Within 24 h of collection, the tissue was homogenized and immediately extracted using 50 mg mL^−1^ 75% methanol for 1 h at 4 °C in the dark. After centrifugation, a mixture of 100 µL of supernatant and methanol was tested by HPLC to separate GSH and GSSG using a Waters ACQUITY UPLC I‐CLASS ultra‐HPLC system (Waters Corporation, Milford, USA). A Waters UPLC HSS T3 column (1.8 µm, 2.1 mm × 100 mm) was used at a temperature of 40 °C with a mobile phase consisting of water containing 0.1% formic acid (phase A) and acetonitrile (phase B), flowing at a rate of 0.5 mL min^−1^ with sample volume set to be at least 5 mL. Glutathione molecules were detected using an ultraviolet detector at a wavelength of 200 nm, which can detect low levels, and quantification was performed using the external standard method. Linear regression was performed by preparing, separating, and determining standard solutions containing GSH and GSSG under the selected chromatographic conditions. The chromatograms and peak areas were recorded for analysis. The HPLC software package program TargetLynx was used for external standardization, with an 8‐minute analysis time for glutathione. During the analysis, GSH eluted from the HPLC column between 1.55 and 1.77 min, whereas GSSG eluted between 0.97 and 1.01 min. Separate linear regressions were generated for both GSH and GSSG, owing to the ease of GSH oxidation. In addition, the linear regression for each analyte covered a wide concentration range (0–1000 ng g^−1^), with the *y*‐axis representing the observed chromatographic peak area and the *x*‐axis representing the analyte concentration. Linear regression using GraphPad Prism 9.0 yielded the following equations: For GSH: *Y* = 19644*X + 59741 with *R* ^2^ = 0.9999; for GSSG: *Y* = 1171*X − 20107 with *R* ^2^ = 0.9948. A standard solution containing GSH and GSSG was prepared and injected following the linear regression method, with subsequent recording of the chromatographic peak area for incorporation into the standard curve. The recovery rate was determined by calculating the ratio of the measured to the added amounts. The results demonstrated recovery rates exceeding 93.9% and 86.3% for GSH and GSSG, respectively. The results are shown in Table S2 (Supporting Information).

The relative standard deviation (RSD = STDEV/AVERAG) was calculated based on the results of the four daily measurements. The RSD for GSH was ≤1.5%, and the RSD for GSSG was ≤1.7%. The results are shown in Table S2 (Supporting Information). Therefore, this assay can measure both GSH and GSSG and is sensitive to small amounts of GSSG.

The total glutathione, GSH, and GSSG levels were assessed, and the GSH/GSSG ratio was calculated. Changes in GSSG levels are a highly sensitive method for evaluating the redox status, and the conversion of GSH to GSSG is widely acknowledged as a reliable indicator of oxidative stress. In this study, total glutathione was calculated by summing the values of GSH and two times the value of GSSG (ng g^−1^). Redox status was evaluated based on the ratio between GSH and GSSG.

### Western Blotting

Tissues were homogenized on ice using RIPA lysis buffer, followed by centrifugation at 14 000*g* for 20 min at 4 °C. The resulting supernatant was stored at −80 °C for future analysis. The protein concentration was quantified using the bicinchoninic acid (BCA) assay (Beyotime, Shanghai, China), each line with loading of 20 µg total protein. Proteins were then separated by 12.5% sodium dodecyl sulfate‐polyacrylamide gel electrophoresis (SDS‐PAGE) and subsequently transferred to a 0.22 µm polyvinylidene difluoride (PVDF) membrane. The membranes were blocked with a solution of 10% skimmed milk powder in Tris‐buffered saline containing 0.1% Tween 20 (TBST) for 1 h at room temperature.

The membranes were incubated overnight at 4 °C with primary antibodies specific to *D2HGDH*, GAPDH, CAT, SOD, Prdx6, and GPX4, respectively, as detailed in Table S7 (Supporting Information). Following this incubation, the membranes were washed three times with TBST, with each wash lasting 10 min. Subsequently, they were incubated at room temperature for 1 h with horseradish peroxidase (HRP)‐conjugated secondary antibodies, either anti‐mouse or anti‐rabbit. After three additional washes with TBST, the protein bands were developed using an enhanced chemiluminescence reagent (Thermo Fisher Scientific, Marina) and visualized with the Fusion FX5 image analysis system (Vilber Lourmat, Marne‐la‐Vallée, France). The relative levels of the proteins were quantified by normalizing the detected signals to the levels of GAPDH, utilizing the Quantity One software (Bio‐Rad, Hercules, CA).

### SOD/CAT/GPx/aiPLA2 Activity Assays

Fresh brain tissue samples were collected and homogenized per gram of tissue with 10 mL of cold 20 × 10^−3^ m HEPES buffer and centrifuged at 1500×*g* for 5 min at 4 °C. Similarly, adherent cells were collected by centrifugation (1000–2000×*g*, incubated at 4 °C for 10 min) per well of a six‐well with 10 mL of cold 20 × 10^−3^ m HEPES buffer. The supernatant was carefully removed and then stored on ice at −80 °C to maintain enzyme activity if the samples were not analyzed on the same day. The activities of the four enzymes were measured under frozen conditions (12 °C), while the enzyme activities in control tissues were assessed under normal conditions (28 °C). Briefly, the activities of SOD, CAT, GPx, and aiPLA2 were measured using appropriate kits following the manufacturer's instructions. Absorbance values were recorded at wavelengths of 450, 540, 340, and 405 nm. All kits were obtained from Cayman Chemicals (Ann Arbor, MI).

### Whole‐Cell Patch‐Clamp Recordings

Coronal slices containing hippocampus were obtained from 8 to 10 weeks old male C57BL/6J mice infected with AAV. All electrophysiological experiments were conducted by researchers who were blinded to the experimental conditions. Mice were anesthetized by intraperitoneal injection of 1.0% pentobarbital, followed by transcardial perfusion with cold (0–4 °C) slice cutting solution containing (in mm): 110 choline chloride, 2.5 KCl, 1.25 NaH_2_PO_4_, 25 NaHCO_3_, 0.5 CaCl_2_, 7 MgCl_2_, 10 d‐glucose (300 mOsm, pH 7.4, saturated with 95% O_2_/5% CO_2_). Brains were removed and sectioned in the ice‐cold slice cutting solution with a vibratome (Leica VT1200S, Germany) to obtain 300 µm coronal slices. Slices containing hippocampus were incubated at 37 °C for 20 min in artificial cerebrospinal fluid (aCSF) containing (in mm): 140 NaCl, 5 KCl, 1.8 CaCl_2_, 1.2 MgCl_2_, 10 d‐glucose (300 mOsm, pH 7.4, saturated with 95% O_2_/5% CO_2_), and then stored at room temperature for 1 h before use. A coronal slice containing hippocampus was placed in a submersion chamber maintained at room temperature and perfused at 3 mL min^−1^ aCSF with 95% O_2_/5% CO_2_. Neurons were visualized with video‐assisted infrared differential interference contrast imaging under a water immersion objective (40×, 0.8 numerical aperture) on an upright SliceScope Pro 1000 microscope (Scientifica) with an infrared IR‐1000 CCD camera (DAGE‐MTI). In addition, transfected hippocampal CA1 pyramidal neurons were identified using EGFP fluorescence and from a pyramidal somatic shape. All recordings were done with patch pipettes with a resistance between 3 and 6 MΩ. Recordings were performed in the whole‐cell configuration using a Multiclamp 700B amplifier (Axon, USA) and Digidata 1440A (Molecular Devices Co., San Jose, CA) with 10 kHz digitization (2 kHz low‐pass Bessel filter) for current‐clamp recordings and 50 kHz digitization (10 kHz low‐pass Bessel filter) for voltage‐clamp recordings. Data were acquired and analyzed using pCLAMP 10.7 software (Molecular Devices Co., San Jose, CA). Series resistance changes were monitored throughout the experiment, and neurons were discarded if the series resistance surpassed 25 MΩ or changed by >20%. Neurons with unstable resting potential or potential >−50 mV were discarded.

For current‐clamp recordings, the internal solution containing (in mm): 60 K_2_SO4, 60 NMG, 40 HEPES, 4 MgCl_2_, 0.5 BAPTA, 12 phosphocreatine, 2 Na_2_ATP, and 0.2 Na_3_GTP (290 mOsm, pH 7.3). D‐APV (50 × 10^−6^ m), DNQX (20 × 10^−6^ m), and picrotoxin (100 × 10^−6^ m) were added to the extracellular fluid to block synaptic transmission. After successfully rupturing the cell membrane, the whole‐cell recording form was obtained, which was stable for 3 min and then switched to *I* = 0 mode to record the resting membrane potential of the cell. A current step protocol was used to evoke action potentials by injecting 500 ms long depolarizing current steps of increasing amplitude from −30 to 160 pA (△10 pA).

For voltage‐clamp recordings, the internal solution used to record mEPSCs and PPR containing (in mm): 130 CsMeSO_4_, 10 HEPES, 10 CsCl, 4 NaCl, 1 MgCl_2_, 1 EGTA, 5 NMG, 5 MgATP, 0.5 Na_3_GTP, and 12 phosphocreatine (280–300 mOsm, pH 7.3). The mIPSCs were recorded by using internal solution that contains (in mm): 100 CsCl, 10 HEPES, 1 MgCl_2_, 1 EGTA, 30 NMG, 5 MgATP, 0.5 Na_3_GTP, and 12 phosphocreatine (280–300 mOsm, pH 7.3). The mEPSCs recording requires the addition of PTX (100 × 10^−6^ m) to the extracellular fluid to block GABA receptors, and TTX (1 × 10^−6^ m) to block TTX‐sensitive sodium channels. The mIPSCs were recorded by the addition of TTX (1 × 10^−6^ m) to the extracellular fluid to block TTX‐sensitive sodium channels, DNQX (20 × 10^−6^ m) to block the AMPA receptor, and D‐APV (50 × 10^−6^ m) to block the NMDA receptor. CA1 pyramidal neurons were clamped at −70 mV and recorded for 5 min. For PPR recordings, picrotoxin (100 × 10^−6^ m) were added to the extracellular fluid to block inhibitory synaptic transmission and CA1 pyramidal neurons were clamped at a holding potential of −70 mV to record Schaffer collateral evoked EPSCs. Paired pulses stimulation was separated by intervals of 50 ms.

### Whole‐Cell Patch‐Clamp Data Analysis

Data were analyzed offline utilizing pClamp 10.7 (Molecular Devices). The properties of single action potential were evaluated through the first action potential stimulated by threshold current. Action potential latency was defined as the time interval between the initiation of the current injection and the achievement of the action potential threshold. The amplitude of an action potential is characterized by the voltage difference between the threshold and the peak value of the action potential. Action potential half‐width was measured as the duration of the action potential at the voltage halfway between the action potential threshold and peak. AHP amplitude was determined as the minimum voltage following the action potential peak subtracted from the action potential threshold. AHP time was determined as the time between action potential threshold and the negative peak of AHP. For the analysis of spike trains, Clampfit 10.7 was used to detect action potentials.

The amplitudes and frequencies of mEPSCs and mIPSCs were detected by MiniAnalysis 6.0 (Synaptosoft). The detection parameters as follows: threshold, 10 pA; period to search a local maximum, 8 ms; time before a peak for baseline, 4 ms; period to search a decay time, 6 ms; fraction of peak to find a decay time, 0.68; period to average a baseline, 1 ms; area threshold, 8 ms pA; number of points to average for peak, 1; direction of peak, negative. The frequency of miniature postsynaptic currents (mEPSCs or mIPSCs) refers to the number of events occurring per unit of time. The amplitude of miniature postsynaptic currents (mEPSCs or mIPSCs) is measured as the peak value of the average current trace from baseline to peak. The PPR EPSC amplitudes were calculated by measuring the peak for the current. The ratio of the second EPSC's amplitude to the first EPSC's amplitude (P2/P1) was utilized for EPSC‐PPR statistical analysis.

### Quantification and Statistical Analysis

Experimental data were presented as mean ± standard error (SEM). For independent samples, comparisons between groups were conducted using either the Student's *t*‐test or the Mann–Whitney *U* test. Comparisons involving three or more groups were performed using one‐way ANOVA, two‐way ANOVA, the Kruskal–Wallis test, and Post hoc test. Quantitative western blotting and immunofluorescence analyses were conducted using ImageJ software. Statistics for mEPSCs and mIPSCs were analyzed using MiniAnalysis 6.0. Action potentials and EPSC‐PPR were calculated using pClamp 10.7. LFPs analysis and spectral imaging were performed with NeuroExplorer 5. Histograms were generated using GraphPad Prism (version 9.0). Bioinformatics statistical computing and the production of corresponding graphics (volcano maps, heat maps, or other graphics) were performed using R version 4.2.3. *P* < 0.05 was considered statistically significant and was indicated as *P < 0.05, **P < 0.01, ***P < 0.001, or ****P < 0.0001 in the summary graphs.

## Conflict of Interest

The authors declare no conflict of interest.

## Author Contributions

Z.Z., H.Z., and P.Z. contributed equally to this work. Z.Z., X.T., Y.M., and P.K. conceived the project and designed the experiments. Z.Z., H.Z., P.Z., R.L., J.Z., J.L., D.H., R.H., F.T., J.L., D.X., and C.Z. performed the experiments. Z.Z., H.Z., and P.Z. analyzed the data. Z.Z., X.T., Y.M., and P.K. wrote the manuscript. All authors revised and approved the final version of the manuscript.

## Supporting information



Supporting Information

Supplemental Video 1

## Data Availability

The data that support the findings of this study are available from the corresponding author upon reasonable request.
