# Supplementary material for: D2HGDH Deficiency Regulates Seizures through GSH/Prdx6/ROS‐Mediated Excitatory Synaptic Activity
Source: Adv Sci (Weinh). 2024 Dec 30;12(13):2404488. doi: 10.1002/advs.202404488 (PMC11967838; doi:10.1002/advs.202404488)
Supplement: Supplementary file 1 — Supporting Information [file ADVS-12-2404488-s001.docx]

Supporting Information

***D2HGDH* Deficiency Regulates Seizures Through GSH/Prdx6/ROS-mediated Excitatory Synaptic Activity**

*Zhijuan Zhang, Hui Zhang, Peng Zhang, Rong Li, Jinyu Zhou, Jiyuan Li , Danmei Hu , Rui Huang, Fenglin Tang, Jie Liu, Demei Xu, Chenlu Zhang, Xin Tian* ＆ Yuanlin Ma* ＆ Patrick Kwan^*^*

**Figure S1-6**

**Table S1-7**


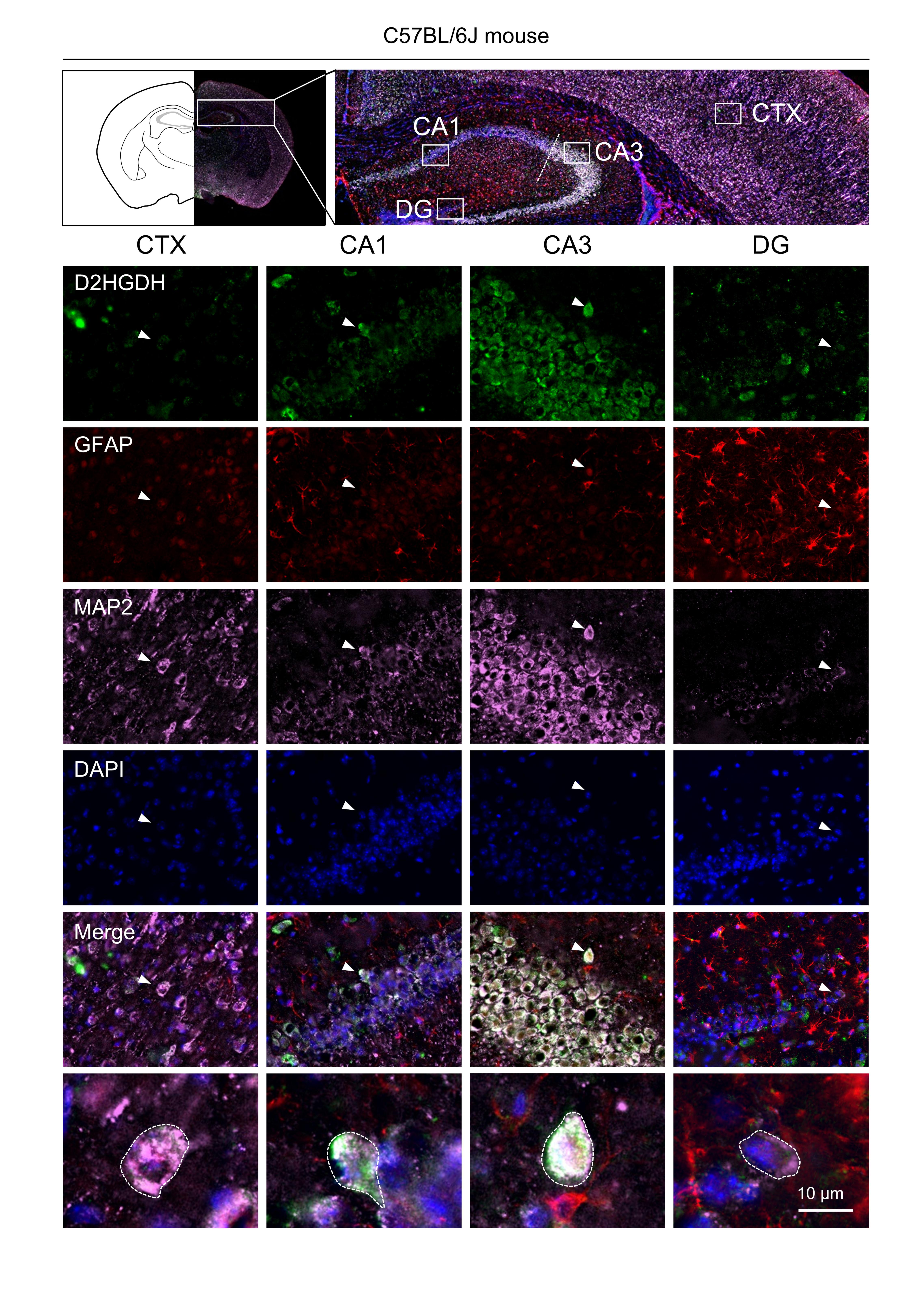


**Figure S1: Localization of D2HGDH in C57BL/6J Mice.**

Co-staining of D2HGDH, GFAP, and MAP2 in naïve C57BL/6J mice (hippocampus, including CA1, CA3, DG, and cortex); scale bar as indicated.





**Figure S2:** **Effects of upregulation and downregulation of D2HGDH on neuronal morphology in the hippocampus.**

(A) Timeline of viral interventions in four AAV groups (AAV-Sh-con vs. AAV-Sh-D2HGDH and AAV-con vs. AAV-D2HGDH). (B) Efficiency of viral infection in C57BL/6J hippocampal neurons. (C) Zoomed tracking of EGFP-infected neurons in the hippocampal CA1, CA3, and DG regions; scale bar as indicated. (D) Zoomed tracking of EGFP-infected neuronal dendritic spines in the hippocampal CA1, CA3, and DG regions; scale bar as indicated. (E) Dendritic spines were morphologically classified as mature (stubby, length/width ratio < 1; mushroom-shaped, width > 0.6 μm; cup-shaped, two or more heads) or immature (filopodia, length > 2 μm; thin, length < 1 μm). (F-H) Statistics for the proportions of mature dendritic spines in CA1, CA3, and DG; n=50 per group. (I-K) Statistics for the lengths of dendritic spines in CA1, CA3, and DG were analyzed using one-way ANOVA; n=37 per group. (L) Statistics for the number of spines per 20 μm were analyzed using one-way ANOVA; n=12 per group. (M) Co-staining of SYN, VGLUT, and MAP2 in C57BL/6J cultured neurons (AAV-Sh-con vs. AAV-Sh-D2HGDH); scale bar as indicated. (N) Quantized data for VGLUT mean pixel intensity (SYN & VGLUT) were analyzed using Student’s t-test. n=10 per group.


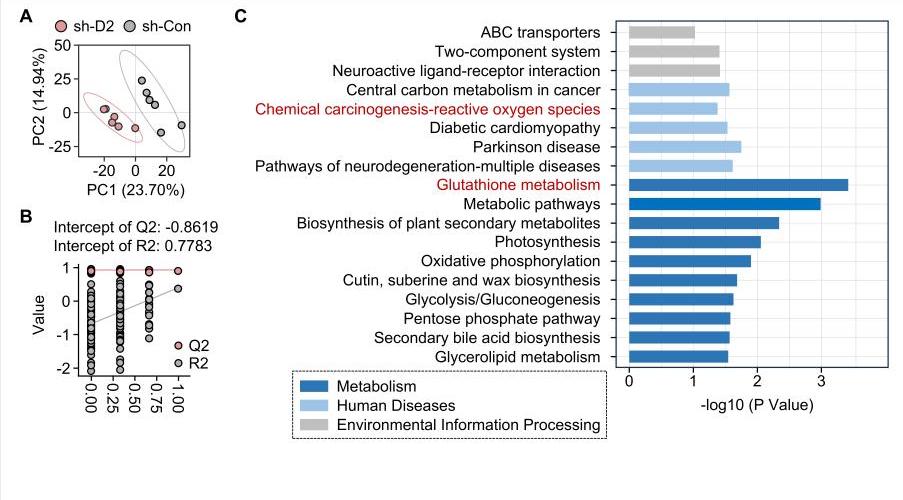


**Figure S3: Differential metabolite analysis between D2HGDH downregulation and control groups.**

(A) Principal Component Analysis: The abscissa (X) represents the first principal component (PC1), while the ordinate (Y) represents the second principal component (PC2) in the comparison of AAV-Sh-D2HGDH versus AAV-Sh-con. (B) Partial Least Squares Discriminant Analysis (PLS-DA): Q2 and R2 values greater than 0.5 indicate a favorable outcome (Intercept of Q2: -0.8619; Intercept of R2: 0.7783) for AAV-Sh-D2HGDH compared to AAV-Sh-con. (C) KEGG-based identification of differentially abundant metabolites: chemical carcinogenesis-ROS, P=0.03571; glutathione metabolism, P=0.00025.


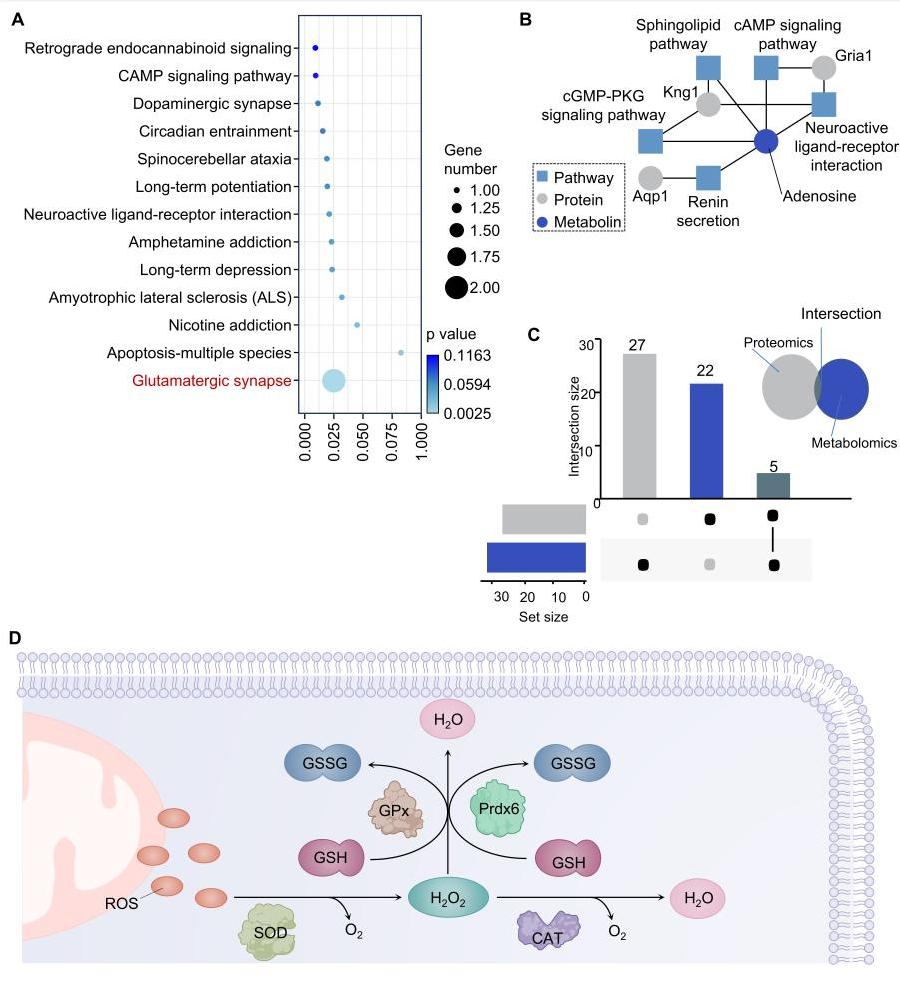


**Figure S4:** **Intracellular glutathione redox reactions and synaptic glutamate function highlight the potential role of D2HGDH in epilepsy.**

(A) Bubble chart illustrating the KEGG-based identification of differentially abundant proteins using TMT in the AAV-Sh-D2HGDH and AAV-Sh-con groups. (B) Overview of pathways, differential metabolites, and differential proteins derived from the combined analysis. (C) UpSet plots representing the combined analysis of pathway-based metabolomics and proteomics. (D) Intracellular ROS metabolism involves a glutathione redox process that includes SOD, CAT, Prdx6, and GPx.


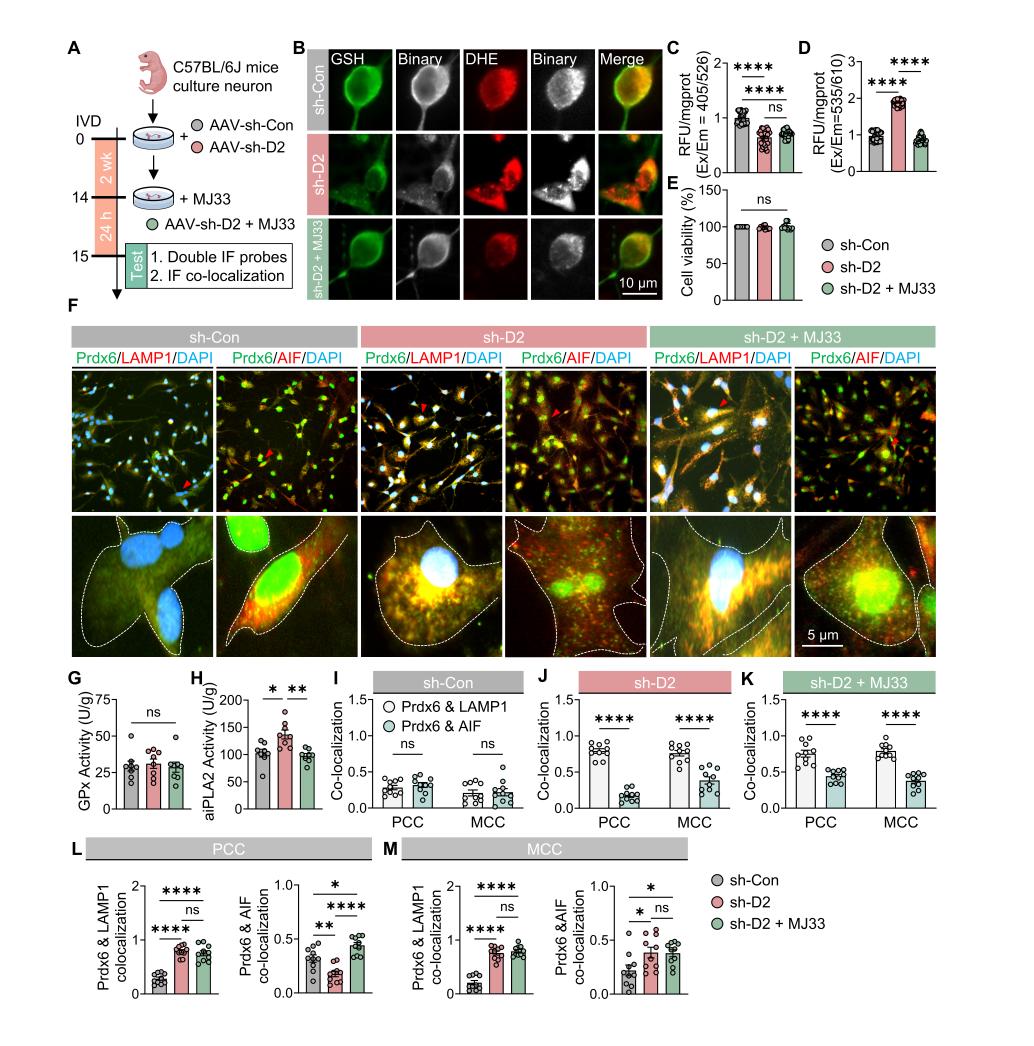


**Figure S5: The aiPLA2 inhibitor MJ33 counteracts the increase in ROS levels induced by D2HGDH knockdown.**

(A) Timeline of C57BL/6J cultured neurons *in vitro* (AAV-Sh-con, AAV-Sh-D2HGDH, and AAV-Sh-D2HGDH+MJ33). (B) Dual probes for GSH (green) and DHE (red) were utilized to trace neurons in vitro; scale bar as indicated. (C) Immunofluorescence quantitative analysis of GSH using one-way ANOVA, n=37 per group. (D) DHE immunofluorescence quantitative analysis of ROS using one-way ANOVA, n=37 per group. (E) CCK-8 assay to quantify neuronal activity using one-way ANOVA, n=10 per group. (F) Representative immunofluorescence images showing the co-localization of Prdx6 (green) with LAMP1 (lysosomal marker, red) and AIF (mitochondrial marker, red) across three groups (AAV-Sh-con, AAV-Sh-D2HGDH, and AAV-Sh-D2HGDH+MJ33). scale bar as indicated. (G) Quantitative data for GPx activity analyzed using one-way ANOVA in the three groups (n=8 per group). (H) Quantitative data for aiPLA2 activity analyzed using one-way ANOVA in the three groups (n=8 per group). (I–K) Quantitative co-localization analysis conducted using JACoP (ImageJ), with PCC and MCC indicating co-localization of Prdx6 with LAMP1 and AIF, defined by PCC or MCC values ≥ 0.5, in the AAV-Sh-con group (I), AAV-Sh-D2HGDH group (J), and AAV-Sh-D2HGDH+MJ33 group (K); n=10 per group, analyzed by two-way ANOVA. (L) Quantitative PCC analysis of Prdx6 with LAMP1 and AIF in the AAV-Sh-con, AAV-Sh-D2HGDH, and AAV-Sh-D2HGDH+MJ33 groups, analyzed using one-way ANOVA; n=10 per group. (M) Quantitative MCC analysis of Prdx6 with LAMP1 and AIF in the AAV-Sh-con, AAV-Sh-D2HGDH, and AAV-Sh-D2HGDH + MJ33 groups, analyzed using one-way ANOVA; n=10 per group.


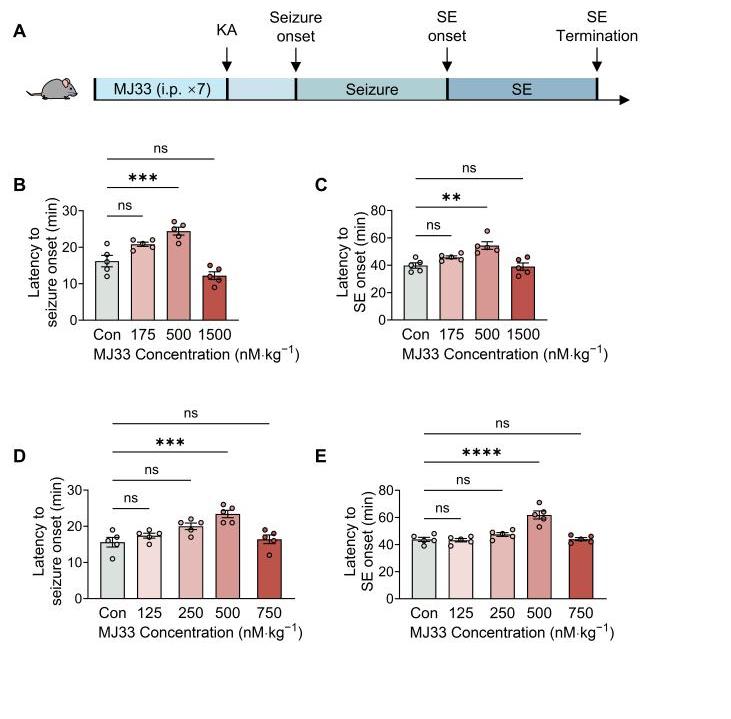


**Figure S6: MJ33 dose-screening in KA-induced acute seizure mice by attenuating seizure onset.**

(A) The timeline and experimental procedure for the KA-induced acute seizure model, following injection with MJ33, involved dividing the animals into five groups based on the concentration of MJ33. B-C Each group received an intraperitoneal injection of MJ33 for 7 days at the following concentrations: 0, 175, 500, and 1500 nM·kg⁻¹. D-E Each group received an intraperitoneal injection of MJ33 for 7 days at the following concentrations: 0, 125, 250, 500, and 750 nM·kg⁻¹. (B) Quantized data for latency to seizure onset following KA injection were analyzed using a one-way ANOVA (n=5 per group). (C) Quantized data for latency to SE onset following KA injection were analyzed using a one-way ANOVA (n=5 per group). (D) Quantized data for latency to seizure onset following KA injection were analyzed using a one-way ANOVA (n=5 per group). (E) Quantized data for latency to SE onset following KA injection were analyzed using a one-way ANOVA (n=5 per group).

**Table S1-7**

**Table S1**: The clinical characterization of TLE patients and TBI patients.

| Sex | Age  (years) | Duration  (years) | Preoperative ASM  consumption | Side of resected temporal lobe | Pathological  diagnosis |
| --- | --- | --- | --- | --- | --- |
| M | 31 | 5 | CBZ, TPM, CZP | L | NL, G |
| F | 28 | 10 | CBZ, PB, LTG, LEV | R | NL, G |
| F | 27 | 9 | CBZ, TPM, CZP | L | NL, G |
| M | 26 | 0 | None | R | N |
| F | 22 | 0 | None | L | N |
| F | 27 | 0 | None | L | N |

ASM, anti-seizure medication; E, epilepsy; C, control; M, male; F, female; CBZ, carbamazepine; TPM, topiramate; CZP, clonazepam; PB, phenobarbital; LTG, lamotrigine; LEV, levetiracetam; L, left; R, right; NL, neuron loss; G, gliosis; N, relative normal.

**Table S2:** GSH, GSSG concentrations and GSH/GSSG ratios in AAV-sh-Con vs AAV-sh-D2HGDH groups.

| **Groups** | **AAV-sh-Con** | | | **AAV-sh-D2HGDH** | | |
| --- | --- | --- | --- | --- | --- | --- |
|  | **GSH^*^** | **GSSG^#^** | **GSH/GSSG** | **GSH*** | **GSSG^#^** | **GSH/GSSG** |
| N = 1 | 942.8812 | 143.5441 | 6.568581 | 1886.484 | 751.662 | 2.50975 |
| N = 2 | 1500.212 | 297.3986 | 5.044449 | 443.7362 | 325.512 | 1.363192 |
| N = 3 | 1424.468 | 430.4264 | 3.309434 | 1365.032 | 718.222 | 1.900571 |
| N = 4 | 918.1544 | 164.4135 | 5.584422 | 776.9906 | 630.929 | 1.231502 |
| N = 5 | 1403.672 | 201.9322 | 6.951204 | 945.9525 | 1052.195 | 0.899028 |
| N = 6 | 1149.85 | 189.6501 | 6.063009 | 399.2383 | 213.8044 | 1.867307 |
| N = 7 | 1488.639 | 280.1805 | 5.313142 | 248.0414 | 121.2083 | 2.046406 |
| N = 8 | 2346.84 | 312.8299 | 7.501968 | 788.8206 | 154.8905 | 5.092762 |
| N = 9 | 1814.949 | 191.9364 | 9.455992 | 552.6065 | 177.5261 | 3.112819 |
| N=10 | 1968.169 | 177.3459 | 11.09791 | 1408.504 | 248.168 | 5.675607 |
| N=11 | 2446.456 | 265.186 | 9.225434 | 1103.606 | 328.1413 | 3.363205 |
| N=12 | 1615.764 | 222.6056 | 7.258416 | 1013.908 | 249.734 | 4.059953 |
| N=13 | 1383.139 | 176.7924 | 7.823519 | 623.0852 | 187.7683 | 3.318372 |
| N=14 | 1423.155 | 155.0862 | 9.17654 | 1002.577 | 287.9434 | 3.481856 |
| N=15 | 1958.891 | 170.8622 | 11.46474 | 797.2933 | 217.0671 | 3.673027 |
| N=16 | 1571.429 | 205.8576 | 7.633573 | 788.8206 | 154.8905 | 5.092762 |

| **Linear regression of GSH** | | | | |
| --- | --- | --- | --- | --- |
| Added Conc.^#^ (ng/g) | Peak Area | Measured Conc. (ng/g) | Rate of Recovery^&^ | **** |
| 0.5 | 24037.459 | 0 | 0 |  |
| 1 | 43359.371 | 0 | 0 |  |
| 5 | 200525.625 | 5.258069325 | 1.051613865 |  |
| 10 | 281370.250 | 9.394317787 | 0.939431779 |  |
| 50 | 1076383.700 | 50.06954347 | 1.001390869 |  |
| 100 | 2109856.200 | 102.945032 | 1.02945032 |  |
| 200 | 3937353.500 | 196.4451588 | 0.982225794 |  |
| 500 | 9772717.000 | 494.9994945 | 0.989998989 |  |
| 1000 | 19758244.000 | 1005.888384 | 1.005888384 |  |
| **Linear regression of GSSG** | | | | |
| Added Conc. (ng/g) | Peak Area | Measured Conc. (ng/g) | Rate of Recovery |  |
| 0.5 | 185.337 | 0 | 0 |  |
| 1 | 278.110 | 1.131412853 | 1.1314128533 |  |
| 5 | 2649.499 | 6.214797147 | 1.242959429 |  |
| 10 | 11832.105 | 8.62822785 | 0.862822786 |  |
| 50 | 23012.539 | 45.67691852 | 0.91353837 |  |
| 100 | 53204.309 | 96.24887054 | 0.962488705 |  |
| 200 | 216134.898 | 203.27627 | 1.01638135 |  |
| 500 | 518734.031 | 504.0706523 | 1.008141305 |  |
| 1000 | 1178577.600 | 998.6418064 | 0.998641806 |  |
| Conc.: Concentration; ^&^Rate of Recovery = Measured Conc./Added Conc. | | | | |

|  | **GSH** | **GSSG** |
| --- | --- | --- |
| QC1 | 168.4877218384 | 682.7196632578 |
| QC2 | 163.1411693310 | 656.1122991523 |
| QC3 | 167.9899952896 | 662.6303509738 |
| QC4 | 165.1338906972 | 666.9383711271 |
| RSD#% | 1.5% | 1.7% |
| ^#^RSD: Relative Standard Deviation = STDEV/AVERAG | | |

**Table S3:** Metabolic KEGG pathways in AAV-sh-Con vs AAV-sh-D2HGDH groups.

| **Gene** | **log2FC** | **Function** |
| --- | --- | --- |
| ABC transporters | 1.07637455 | Environmental Information Processing |
| Two-component system | 1.478230134 | Environmental Information Processing |
| Neuroactive ligand-receptor interaction | 1.48637626 | Environmental Information Processing |
| Central carbon metabolism in cancer | 1.632280925 | Human Diseases |
| Chemical carcinogenesis - reactive oxygen species | 1.447136669 | Human Diseases |
| Diabetic cardiomyopathy | 1.609671072 | Human Diseases |
| Parkinson disease | 1.836982716 | Human Diseases |
| Pathways of neurodegeneration - multiple diseases | 1.694699957 | Human Diseases |
| Glutathione metabolism | 3.588188839 | Metabolism |
| Metabolic pathways | 3.135661216 | Metabolism |
| Biosynthesis of plant secondary metabolites | 2.456652834 | Metabolism |
| Photosynthesis | 2.155797694 | Metabolism |
| Oxidative phosphorylation | 1.99370394 | Metabolism |
| Cutin, suberine and wax biosynthesis | 1.767853259 | Metabolism |
| Glycolysis/Gluconeogenesis | 1.708361675 | Metabolism |
| Pentose phosphate pathway | 1.656161543 | Metabolism |
| Secondary bile acid biosynthesis | 1.644053621 | Metabolism |
| Glycerolipid metabolism | 1.620825572 | Metabolism |

**Table S4:** Transcriptic KEGG pathways in AAV-sh-Con vs AAV-sh-D2HGDH groups.

| **Term_ID** | **Term** | **bg_pro**  **num.** | **bg_term**  **num.** | **fg_pro**  **num.** | **fg_term**  **num.** | **Ratio** | **p value** | **Enrichment** | **FDR** | **IDs** | **Gene_**  **symbol** |
| --- | --- | --- | --- | --- | --- | --- | --- | --- | --- | --- | --- |
| path:mmu04724 | Glutamatergic synapse | 2701 | 79 | 3 | 2 | 0.025316 | 0.002487 | 2.604385 | 0.032326 | GRIA1_MOUSE,  A0A1C7ZMY3_MOUSE | Gria1,  Shank2 |
| path:mmu04215 | Apoptosis - multiple species | 2701 | 12 | 3 | 1 | 0.083333 | 0.013274 | 1.876993 | 0.08186 | A0A087WR00_MOUSE | Bok |
| path:mmu05033 | Nicotine addiction | 2701 | 22 | 3 | 1 | 0.045455 | 0.024246 | 1.615363 | 0.08186 | GRIA1_MOUSE | Gria1 |
| path:mmu05014 | Amyotrophic lateral sclerosis (ALS) | 2701 | 31 | 3 | 1 | 0.032258 | 0.03405 | 1.467877 | 0.08186 | GRIA1_MOUSE | Gria1 |
| path:mmu04730 | Long-term depression | 2701 | 42 | 3 | 1 | 0.02381 | 0.045945 | 1.337766 | 0.08186 | GRIA1_MOUSE | Gria1 |
| path:mmu05031 | Amphetamine addiction | 2701 | 43 | 3 | 1 | 0.023256 | 0.047021 | 1.327709 | 0.08186 | GRIA1_MOUSE | Gria1 |
| path:mmu04080 | Neuroactive ligand-receptor interaction | 2701 | 47 | 3 | 1 | 0.021277 | 0.051318 | 1.289726 | 0.08186 | GRIA1_MOUSE | Gria1 |
| path:mmu04720 | Long-term potentiation | 2701 | 51 | 3 | 1 | 0.019608 | 0.055603 | 1.254901 | 0.08186 | GRIA1_MOUSE | Gria1 |
| path:mmu05017 | Spinocerebellar ataxia | 2701 | 52 | 3 | 1 | 0.019231 | 0.056672 | 1.24663 | 0.08186 | GRIA1_MOUSE | Gria1 |
| path:mmu04713 | Circadian entrainment | 2701 | 64 | 3 | 1 | 0.015625 | 0.069439 | 1.158398 | 0.09027 | GRIA1_MOUSE | Gria1 |
| path:mmu04728 | Dopaminergic synapse | 2701 | 87 | 3 | 1 | 0.011494 | 0.093585 | 1.028792 | 0.110601 | GRIA1_MOUSE | Gria1 |
| path:mmu04024 | cAMP signaling pathway | 2701 | 106 | 3 | 1 | 0.009434 | 0.113214 | 0.946098 | 0.116288 | GRIA1_MOUSE | Gria1 |
| path:mmu04723 | Retrograde endocannabinoid signaling | 2701 | 109 | 3 | 1 | 0.009174 | 0.116288 | 0.934467 | 0.116288 | GRIA1_MOUSE | Gria1 |

**Table S5.1:** List of associative differential metabolites (AAV-sh-Con vs AAV-sh-D2HGDH groups).

| **Level_2** | **Level_1** | **Pathway** | **KEGG** | **Number**  **Compound** | **Number**  **Feature** | **Background** | **p value** | **FDR** | **Compound** | **Feature**  **(Metabolites_ID)** | **Name** |
| --- | --- | --- | --- | --- | --- | --- | --- | --- | --- | --- | --- |
| Signal transduction | Environmental Information Processing | cGMP-PKG signaling pathway | map04022 | 1 | 1 | 10 | 0.004768 | 0.0184 | C00212 | pos-0.904_  267.09641 | Adenosine |
| Signal transduction | Environmental Information Processing | cAMP signaling pathway | map04024 | 1 | 1 | 25 | 0.011881 | 0.0247 | C00212 | pos-0.904_  267.09641 | Adenosine |
| Signal transduction | Environmental Information Processing | Sphingolipid signaling pathway | map04071 | 1 | 1 | 15 | 0.007144 | 0.0214 | C00212 | pos-0.904_  267.09641 | Adenosine |
| Signaling molecules and interaction | Environmental Information Processing | Neuroactive ligand-receptor interaction | map04080 | 1 | 1 | 52 | 0.024572 | 0.039 | C00212 | pos-0.904_  267.09641 | Adenosine |
| Endocrine system | Organismal Systems | Renin secretion | map04924 | 1 | 1 | 17 | 0.008093 | 0.0219 | C00212 | pos-0.904_  267.09641 | Adenosine |

**Table S5.2:** List of associative differential proteins (AAV-sh-Con vs AAV-sh-D2HGDH groups).

| **Protein_ID** | **Entry** | **KO** | **FC** | **log2FC** |
| --- | --- | --- | --- | --- |
| KNG1_MOUSE | mmu:16644 | K03898 | 1.227231 | 0.295407 |
| GRIA1_MOUSE | mmu:14799 | K05197 | 0.765111 | -0.38626 |
| AQP1_MOUSE | mmu:11826 | K09864 | 1.228643 | 0.297066 |

**Table S6:** UpSet Joint analysis.

| **Group** | **Intersection_number** | **Intersection_elements** |
| --- | --- | --- |
| Metabolomics | 22 | map00270, map01100, map07227, map00230, map00250, map01240, map00360, map00590, map04270, map02010, map04923, map05012, map05032, map05034, map00908, map00966, map01110, map01210, map00232, map01060, map01065, map01120 |
| Proteomics | 27 | map05143, map04610, map05144, map00910, map04215, map04724, map04964, map05033, map00983, map05014, map04976, map04918, map03320, map05142, map04730, map05031, map04750, map04720, map05017, map04721, map04713, map05166, map04728, map04360, map04723, map04810, map05200 |
| Proteomics, Metabolomics | 5 | map04080, map04924, map04071, map04022, map04024 |

**Table S7:** Specific information for viral vectors, antibodies, and reagents.

| **Reagent or resource** | **Applications** | **Source** | **Identifier** |
| --- | --- | --- | --- |
| pAAV-hysn-EGFP-3×FLAG-miR30shRNA(D2hgdh)-WPRE | 500 nL per hippocampus *in vivo*; 1 μL per well *in vitro*  Ibid.  Ibid.  Ibid. | OBiO Technology | / |
| pAAV-hysn-EGFP-3×FLAG-miR30shRNA(NC)-WPRE |  |  |  |
| pAAV-hysn-*D2hgdh*-EGFP-WPRE |  |  |  |
| pAAV-hysn-EGFP-WPRE |  |  |  |
| DMEM/F-12 | 1 × | Gibco | Cat# 11320033 |
| Penicillin/streptomycin/neomycin (PSN) | 100 × | Gibco | Cat# 15640055 |
| Poly-D-lysine | 10 × | Sigma-Aldrich (St. Louis, Missouri, USA) | Cat# A3890401 |
| B27 supplement | 50 × | Gibco | Cat# 17504044 |
| Neurobasal^TM^ medium | 1 × | Gibco | Cat# 21103049 |
| GlutaMAX^TM^ supplement | 100 × | Gibco | Cat# 35050061 |
| KA | 1.0 nmol in 50 nl NS *in vivo* | Sigma-Aldrich (St. Louis, Missouri, USA) | Cat# 487-79-6 K0250 |
| PTZ | 35 mg⋅kg^−1^ *in vivo* | Sigma-Aldrich (St. Louis, Missouri, USA) | Cat# P6500 |
| MJ33 | 500 nM⋅kg^−1^ *in vivo*; 20 μM *in vitro* | Sigma-Aldrich (St. Louis, Missouri, USA) | Cat# M3315 |
| GSH | 50 mg⋅ml^−1^ | NIFDC (Beijing, China) | Cat# 140706-202004 |
| GSSG | 50 mg⋅ml^−1^ | NIFDC (Beijing, China) | Cat# 140746-201703 |
| D2HGDH pAb | WB: 1:1000, IF: 1:100 | Proteintech (Wuhan, China) | Cat# 13895-1-AP |
| GAPDH mAb | WB: 1:1000 | ZENBIO (Chengdu, China) | Cat# 390035 |
| Prdx6 pAb | WB: 1:1000; IF: 1:100 | ZENBIO (Chengdu, China) | Cat# 381769 |
| SOD pAb | WB: 1:1000 | ThermoFisher Scientific (Waltham, USA) | Cat# PA5-27240 |
| CAT pAb | WB: 1:1000 | ThermoFisher Scientific (Waltham, USA) | Cat# PA5-29183 |
| GPX4 mAb | WB: 1:1000 | ThermoFisher Scientific (Waltham, USA) | Cat# MA5-32827 |
| LAMP1 mAb | IF: 1:100 | Abcam (Cambridge, UK) | Cat# ab302639 |
| AIF mAb | IF: 1:100 | Abcam (Cambridge, UK) | Cat# ab196847 |
| MAP2 mAb | IF: 1:2000 | ZENBIO (Chengdu, China) | Cat# 250035 |
| GFAP mAb | IF: 1:500 | ZENBIO (Chengdu, China) | Cat# 250027 |
| PSD95 mAb | IF: 1:200 | Abcam (Cambridge, UK) | Cat# ab13552 |
| GAD67 mAb | IF: 1:100 | Abcam (Cambridge, UK) | Cat# ab183999 |
| LiveReceptor^TM^ AMPAR | IF: 1μm *in vitro* | Funakoshi (Tokyo, Japan） | Cat# FDV-0018A |
| HRP-conjugated Goat Anti-Mouse IgG (H+L) | WB: 1:5000 | Proteintech (Wuhan, China) | Cat# SA00001-1 |
| HRP-conjugated Goat Anti-Rabbit IgG (H+L) | WB: 1:5000 | Proteintech (Wuhan, China) | Cat# SA00001-2 |
| CoraLite488-conjugated Donkey Anti-Mouse IgG (H+L) | IF: 1:400 | Proteintech (Wuhan, China) | Cat# SA00013-5 |
| CoraLite488-conjugated Donkey Anti-Rabbit IgG (H+L) | IF: 1:400 | Proteintech (Wuhan, China) | Cat# SA00013-6 |
| CoraLite594-conjugated Donkey Anti-Mouse IgG (H+L) | IF: 1:400 | Proteintech (Wuhan, China) | Cat# SA00013-7 |
| CoraLite594-conjugated Donkey Anti-Rabbit IgG (H+L) | IF: 1:400 | Proteintech (Wuhan, China) | Cat# SA00013-8 |
| Cy3-conjugated Goat Anti-Rabbit IgG (H+L) | IF: 1:300 | Servicebio (Wuhan, China) | Cat# GB21303 |
| Cy5-conjugated Goat Anti-rabbit IgG (H+L) | IF: 1:300 | Servicebio (Wuhan, China) | Cat# GB27303 |
| Alexa Fluor^TM^ Plus 555 Goat Anti-Mouse IgG (H+L) | IF: 1:400 | Invitrogen | Cat# A32727 |
| Alexa Fluor^TM^ Plus 405 Goat Anti-Mouse IgG (H+L) | IF: 1:400 | Invitrogen | Cat# A48255 |
| mBBr | 1 μM *in vitro* | MCE | Cat# HY-101899 |
| DHE Assay Kit | / | BJBALB (Beijing, China) | Cat# HR8821 |
| SOD Assay Kit | / | Cayman (Michigan, USA) | Cat# 706002 |
| CAT Assay Kit | / | Cayman (Michigan, USA) | Cat# 707002 |
| GPx Assay Kit | / | Cayman (Michigan, USA) | Cat# 703102 |
| aiPLA2 Assay Kit | / | Cayman (Michigan, USA) | Cat# 765021 |
| CCK8 | / | Beyotime (Shanghai, China) | Cat# C0038 |

NS, Normal saline; NIFDC, National Institutes for Food and Drug Control; IF, Immunofluorescence; WB, Western blotting.
